# New Genetic Insights into Pearl Millet Diversity As Revealed by Characterization of Early- and Late-Flowering Landraces from Senegal

**DOI:** 10.3389/fpls.2017.00818

**Published:** 2017-05-17

**Authors:** Oumar Diack, Ndjido A. Kane, Cecile Berthouly-Salazar, Mame C. Gueye, Baye M. Diop, Amadou Fofana, Ousmane Sy, Hamidou Tall, Leila Zekraoui, Marie Piquet, Marie Couderc, Yves Vigouroux, Diaga Diouf, Adeline Barnaud

**Affiliations:** ^1^Laboratoire Campus de Biotechnologies Végétales, Faculté des Sciences et Techniques, Université Cheikh Anta Diop de DakarDakar, Senegal; ^2^Laboratoire Mixte International Adaptation des Plantes et Microorganismes Associés aux Stress Environnementaux, Centre de Recherche de Bel AirDakar, Senegal; ^3^Laboratoire National de Recherches sur les Productions Végétales, Institut Sénégalais de Recherches Agricoles, Centre de Recherche de Bel AirDakar, Senegal; ^4^Unité Mixte de Recherche Diversité et Adaptation des Espèces, Institut de Recherche pour le DéveloppementMontpellier, France; ^5^Centre d’Etude Régional pour l’Amélioration de l’Adaptation à la Sécheresse, Institut Sénégalais de Recherches AgricolesThiès, Senegal; ^6^Centre National de Recherches Agronomiques de Bambey, Institut Sénégalais de Recherches AgricolesBambey, Senegal; ^7^Centre de Recherches Zootechniques de Kolda, Institut Sénégalais de Recherches AgricolesKolda, Senegal

**Keywords:** millet, *Pennisetum glaucum*, flowering, genetic diversity, SSR, *PgMADS11*, *PgPHYC*

## Abstract

Pearl millet (*Pennisetum glaucum* (L.) R. Br.) is a staple food and a drought-tolerant cereal well adapted to Sub-Saharan Africa agro-ecosystems. An important diversity of pearl millet landraces has been widely conserved by farmers and therefore could help copping with climate changes and contribute to future food security. Hence, characterizing its genetic diversity and population structure can contribute to better assist breeding programs for a sustainable agricultural productivity enhancement. Toward this goal, a comprehensive panel of 404 accessions were used that correspond to 12 improved varieties, 306 early flowering and 86 late-flowering cultivated landraces from Senegal. Twelve highly polymorphic SSR markers were used to study diversity and population structure. Two genes, *PgMADS11* and *PgPHYC*, were genotyped to assess their association to flowering phenotypic difference in landraces. Results indicate a large diversity and untapped potential of Senegalese pearl millet germplasm as well as a genetic differentiation between early- and late-flowering landraces. Further, a fine-scale genetic difference of *PgPHYC* and *PgMADS11* (SNP and indel, respectively) and co-variation of their alleles with flowering time were found among landraces. These findings highlight new genetic insights of pearl millet useful to define heterotic populations for breeding, genomic association panel, or crosses for trait-specific mapping.

## Introduction

Low agricultural productivity causes food insecurity and malnutrition in Sub-Saharan Africa (SSA). This is due to climate variability, increased growth and needs of worldwide population, water demand, intensive exploitation of natural resources and environmental degradation. Therefore, better uses of natural resources could help overcoming some of these constraints and greatly contribute to improving productivity.

Pearl millet (*Pennisetum glaucum* (L.) R. Br.) is a major staple food for many SSA and Asian countries. Pearl millet is highly allogamous and mainly a rainfed crop, covering a wide range of different ecological zones and production systems. However, yield is low and variable, rarely reaching 1000 kg/ha. This is mainly explained by the limited exploitation of genetic resources and availability of improved varieties, but also by low soil fertility, drought, heat and highly variable rainfall (Waddington et al., 2010). Moreover, production is threatened by downy mildew disease, striga parasitic weed and predation by insects (Waddington et al., 2010). Characterization of crops genetic resources is a prerequisite to build up a breeding program for sustainable productivity enhancement.

Senegal is one of the top 10 pearl millet producers in the world (FAOSTAT, 2013), where farmers distinguish two main types of cultivars based on growth duration. Cultivars, called *Souna*, are sensitive to photoperiod, with a short cycle between 65 and 90 days and adapted to low (350–600 mm) rainfall regions. Cultivars, called *Sanio*, are a less photoperiod-sensitive type than Souna, with a long cycle between 120 and 150 days, and are adapted to high (900–1200 mm) rainfall regions. Nationwide, *Souna* type occupies nearly half the area sown to cereals (51%) while *Sanio* type, mainly cultivated in the South, represents about 15% of total millet production (ANSD, 2014).

Investigating genetic diversity and patterns of early- and late-flowering landraces is very important since flowering cycles and photoperiod sensitivity play a crucial role in the adaptation to climatic conditions. It is assumed a direct effect of selection for earliness associated with climate variations (Vigouroux et al., 2011). Genome scans and genetic association mapping have identified several genes tightly linked to adaptive traits of pearl millet in semi-arids areas. A SNP in the *Phytochrome C* locus (*PgPHYC*) and an indel variation in *PgMADS11* gene were associated with flowering time variation, annual rainfall and spike length of pearl millet (Saïdou et al., 2009; Mariac et al., 2011; Vigouroux et al., 2011). From framers point of view, distinction between early- and late-flowering landraces is very clear but it is not always associated with clear genetic differentiation (Dussert et al., 2015). Flowering time in pearl millet is derived from a common domestication event and a strong gene flow between early- and late-flowering landraces is observed (Dussert et al., 2015). The level of gene flow would depend on cycle overlapping, agricultural practices and spatial distribution (Mariac et al., 2006b; Allinne et al., 2007; Lakis et al., 2012).

Because there is a critical need for adapting local agriculture to harsher future conditions, landraces and improved varieties adaptation will mostly rely on standing genetic variation available within the cultivated compartment. Recently, phenotyping (Sy et al., 2015) and genotyping by sequencing (Hu et al., 2015) studies were carried out on a set of Senegalese pearl millet landraces. Sampling was restricted to only one agro-ecological area of Senegal, the Groundnut Basin, and included only nine so-called intermediate-flowering landraces (flower between 75 to 100 days after sowing). Based on phenology, head architecture and grain color, these accessions were classified into three cultivar groups, indicating a morphological diversity between early flowering landraces (Sy et al., 2015). Using 83,875 single nucleotide polymorphisms (SNPs) on the same set of accessions in addition to 252 global accessions, a higher genetic diversity was observed in Senegal accessions compared to millet accessions in India, South and Western Africa (Hu et al., 2015). Any local structure was evidenced, therefore studies of loci that control the cycle length would be necessary to assess more accurately the evolution of cultivated millet varieties (Dussert et al., 2015).

Here, a fine scale sampling strategy and genetic characterization are described that differentiate early- and late-flowering landraces of Senegalese pearl millet. Using highly polymorphic SSRs markers, genetic diversity and population structure of the landraces was assessed. Allelic diversity of *PgPHYC* and *PgMADS11* genes, both linked with flowering time variation and rainfall, was further investigated (Saïdou et al., 2009; Mariac et al., 2011; Vigouroux et al., 2011).

## Materials and Methods

### Plant Materials

Collects were done in 1992 and 1994 in the main areas of millet production in the Groundnut Basin as previously described (Sy et al., 2015). Geographical coordinates of these accessions were partially retrieved (88%) by using village names. Additional collects were done in 2010 and 2014 to cover pearl millet production areas, except the city of Dakar and the eastern-south area where the Niokolo-Koba Wildlife Park is located. Geographical coordinates of these new accessions were recorded using a GPS. As our focus is on local landraces, villages near major roads or markets were avoided. In total, 392 accessions were collected from 316 villages, i.e., 1.24 accessions per village on average. A panel of 404 accessions was analyzed including 12 improved varieties bred locally and widely used by farmers, 306 early flowering landraces (252 villages) and 86 late-flowering landraces (74 villages). Among the 316 villages, 10 villages were sampled with both early- and late- flowering landraces (Supplementary Table S1). Cycles were recorded following farmer interviews.

### DNA Extraction and SSR Genotyping

Five seeds per accession were grown in the greenhouse 3–4 weeks according to sampling date. About 200 mg of leaf sample from one individual per accession were collected and DNA extraction was carried out using the previously described protocol (Mariac et al., 2006a). Twelve highly polymorphic microsatellites distributed throughout the pearl millet genome were used (Supplementary Table S2). These markers have been previously described (Allouis et al., 2001; Qi et al., 2001; Budak et al., 2003; Mariac et al., 2006a). PCR reactions were performed using the Multiplex PCR Kit (Qiagen, Inc) following the recommended protocol. PCR were conducted using a thermal cycler TC-Plus (TECHNE): pre-denaturation of 95°C for 15 min then 35 cycles consisting of a denaturation step at 94°C for 30 s, annealing at 55°C for 90 s, elongation at 72°C for 60 s and a final extension at 60°C for 30 min). Four positive and four negative controls were repeated on each PCR plate. Samples were genotyped on an ABI 3130 Prism^®^ (Applied Biosystems^®^) and read with Genemapper^TM^ software (version 3.7; Applied Biosystems^®^).

### Genetic Diversity and Population Structure Analyses

Genetic diversity, heterozygosities (expected and observed) and F-statistics were calculated using Genalex 6.5 (Peakall and Smouse, 2012). For genetic structure, a principal component analysis (PCA) using the package ade4 (Thioulouse et al., 1997) implemented in R software (R Development Core Team, 2008) was first performed. Then, population structure was investigated using STRUCTURE software 2.3.3 (Pritchard et al., 2000). Analysis was performed with the admixture model (Falush et al., 2003) with K ancestral populations ranging from 1 to 6. We used 500,000 iterations and a burn-in period of 100,000, 10 runs for each *K*-value were performed. The values for the number of clusters (*K*) were assessed according to Evanno et al. (2005) by the (*D.*Δ*K*) criterion and the log-likelihood (Ln P (D | K)) plot. Individuals were assigned to a cluster if their ancestry was higher than 70%, *q* ≥ 0.7.

For spatial analysis of genetic variability, a total of 367 geo-referenced accessions including 281 early- and 86 late-flowering landraces were used. Spatial principal component analysis (sPCA) was performed using the *adegenet* package (Jombart, 2008) with R software. The spatial genetic structure was assessed using spatial autocorrelation analyses of kinship coefficients between individuals (Loiselle et al., 1995) following the standard procedure (Vekemans and Hardy, 2004) implemented in SPAGeDi version 1.2 (Hardy and Vekemans, 2002). Mean multilocus kinship coefficient values, *F*_ij_, i.e., genetic similarity between individuals i and j relative to the mean genetic similarity between random individuals in the sample, were regressed on both the linear (*d*_ij_*)* and the logarithmic (ln(*d*_ij_)) spatial distance between individuals. This distance was calculated as the Euclidian distances using spatial coordinates. The regression slopes *b*_d_ and *b*_Ld_ were jointly assessed. Standard errors for the kinship coefficients were estimated using a jackknife procedure over all loci. We tested the significance of the kinship coefficients and the regression slopes *b*_d_ and *b*_Ld_ estimates by comparing the observed values to those obtained after 10,000 random permutations.

### PgPHYC and PgMADS11 Genotyping

Polymorphisms in both genes were associated with flowering time variation (Saïdou et al., 2009; Mariac et al., 2011; Vigouroux et al., 2011). The panel of accessions was genotyped with *PgPHYC* (Acc numbers FN376885–FN377564) (Vigouroux et al., 2011) and *PgMADS11* (Acc numbers FN552468–FN552522) (Mariac et al., 2011) to test the allelic differences in genotype frequencies between early- and late-flowering landraces.

For *PgPHYC*, a polymorphism at the 5′ of the gene was assessed. A C/G SNP at that position is cleaved by PvuII restriction enzyme and therefore accessions scored as C/C, G/G and C/G according to their digestion pattern (Saïdou et al., 2009).

For *PgMADS11*, an indel polymorphism of 24 bp was assessed as previously reported (Mariac et al., 2011).

Logistic regressions between genotypes, genetic cluster, latitude and longitude for each gene were further performed.

## Results

### Genetic Diversity of Senegalese Germplasm

**FIGURE 1:**
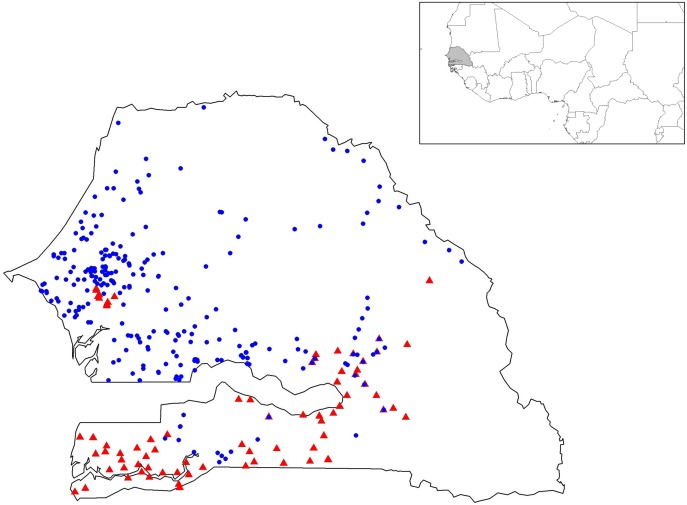
**Geographic positions of the 367 geo-referenced accessions of pearl millet.** Early- and late-flowering landraces are represented by blue circles and red triangles, respectively.

The germplasm collected through this study is the most comprehensive sampling to date of landraces from Senegal (**Figure 1**). Genotyping data revealed a total of 101 alleles with an average of 8.4 alleles per locus (Supplementary Table S2 and Figures S1–S3). The final data set contained only 1.8% of missing data. High levels of genetic diversity characterize both groups (**Table 1**). Early flowering landraces presented the highest level of genetic diversity as measured by observed *H*_Obs_ = 0.481 or expected heterozygosity, *H*_Exp_ = 0.567. In contrast, improved varieties showed the lowest, with *H*_Obs_ = 0.391. For early flowering landraces, only one locus (PSMP2249) was found not to be at Hardy-Weinberg equilibrium (HWE). Two loci were not at HWE (PSMP2249 and PSMP2246) in late-flowering landraces. Groups showed low levels of inbreeding with *F*_IS_ values of 0.160, 0.128, and 0.258 for early and late landraces and improved varieties, respectively. Furthermore, early-, late-flowering landraces and improved varieties present 22, 8 and 1 private alleles, respectively. Low *F*_ST_ differentiation was found between improved varieties and early flowering landraces (0.004), between early- and late-flowering landraces (0.052) and between late-flowering landraces and improved varieties (0.063).

**Table 1:** Genetic diversity statistics for improved varieties, early- and late-flowering landraces.

	*N*b	*N*a	*H*_Obs_	*H*_Exp_	*F*_IS_
Improved varieties	12	4.4	0.350	0.513	0.258
Early flowering landraces	306	7.7	0.446	0.542	0.160
Late-flowering landraces	86	5.8	0.434	0.494	0.128


*Nb, number of accessions; Na, mean number of alleles, H_Obs_, observed heterozygosity; H_Exp_, expected heterozygosity; F_IS_, inbreeding coefficient*.

### Population Genetic Structure

**FIGURE 2:**
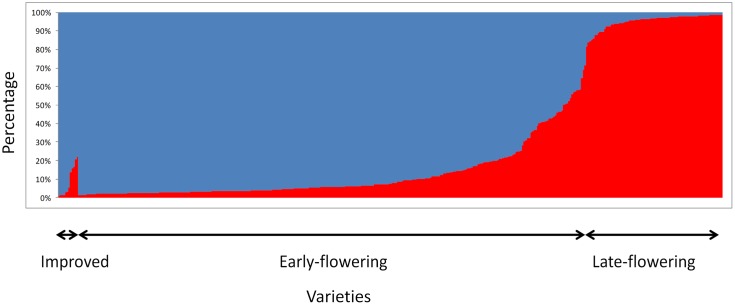
**STRUCTURE results for *K* = 2 based on 404 accessions analyzed with 12 SSRs.** Each bar represents an individual. It shows the proportion of genome belonging to each of the two genetic groups (blue for early flowering accessions and red for late-flowering accessions) identified with STRUCTURE.

Bayesian clustering analyses showed a clear structure between early- and late-flowering landraces (**Figure 2**). The value of the Evanno criterion (ΔK) was the highest for *K* = 2 (Supplementary Figure S1), supporting the evidence of two major clusters. A total of 89% of the early flowering landraces were assigned to a single cluster, and 90% of the late-flowering landraces were assigned to the other cluster. Thirty-four accessions of early- and nine of late-flowering landraces were misassigned (*q* < 0.7), but showed intermediate ancestries. For the 10 villages where both early- and late-flowering landraces were sampled, all accessions of similar phenotype were assigned to their respective cluster.

**FIGURE 3:**
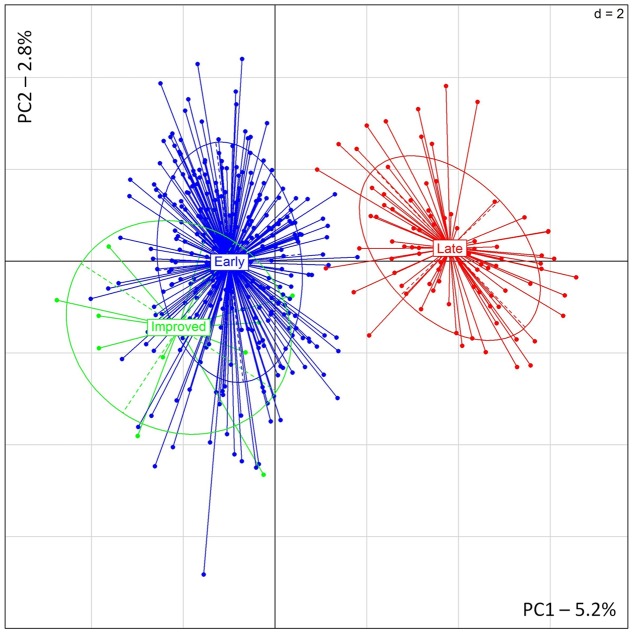
**PCA results obtained for 12 improved, 306 early- and 86 late-flowering landraces accessions**.

Similar results were found with PCA showing a clear distinction between early- and late-flowering landraces (**Figure 3**). The two first principal components explained, respectively, 5.2 and 2.8% of the inertia. PSMP2247 and PSMP2202 showed the highest contribution to the PC1. Two alleles, PSMP2247-199 and PSMP2202-146, showed high frequencies in late-flowering landraces (0.92 and 0.83, respectively). However, removing these two loci did not affect PCA results. Finally, any genetic differentiation was found between improved and early accessions.

### Spatial Analysis of Genetic Variability

**FIGURE 4:**
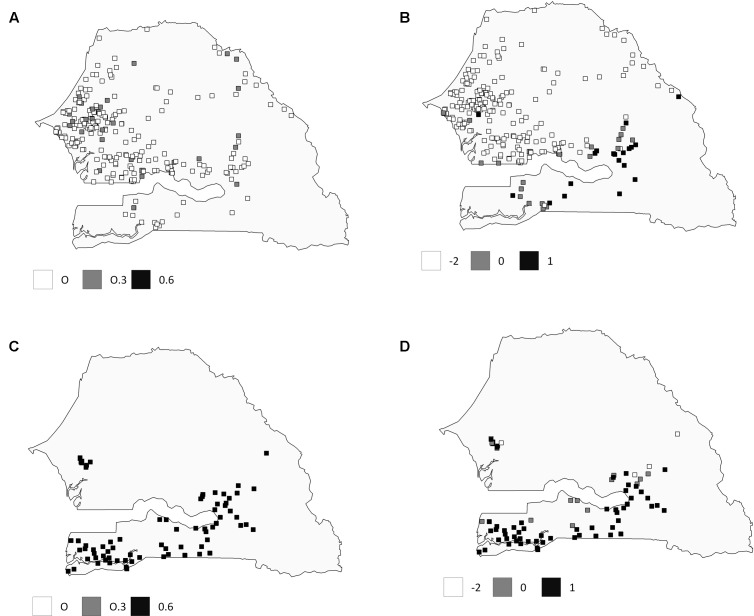
**Comparison of genetic assignment between Structure and SPCA results for early- and late-flowering landraces.** The top row represents values for early flowering landraces: **(A)** with *q*-values obtained with Structure analysis, and **(B)** lagged scores from the sPCA. The bottom row represents values for late-flowering landraces **(C)** with *q*-values obtained with Structure analysis and **(D)** lagged scores from sPCA. Values are depicted in grayscale.

Spatial principal component analysis revealed a more cryptic genetic structure (**Figure 4**). Global structures, i.e., large spatial scale, were significant (*p-value* = 0.0001) with the first principal component, showing a high autocorrelation (Morran’s *I* = 0.50). In contrast, local structures were not significant (*p-value* = 0.77). The first axis of the sPCA identified two clusters. Considering spatial information, early flowering landraces from Southern Senegal showed a more admixed pattern than observed with STRUCTURE, while no differences were observed considering late-flowering landraces. Bayesian and multivariate approaches confirm genetic assignments for late-flowering landraces from Central Senegal.

**FIGURE 5:**
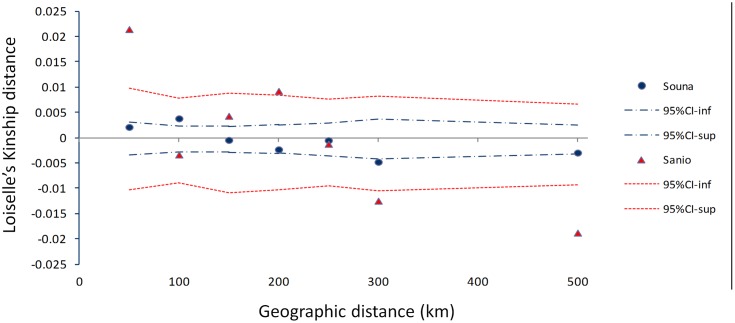
**Isolation by distance pattern for early- (blue dots) and late-flowering (red triangles) landraces as measured by the mean multilocus Loiselle’s kinship genetic distance, plotted against geographical distance (km).** Upper and lower 95% confidence limits are indicated by colored dotted lines. Each point represents averaged Loiselle’s kinship for each class distance.

The pattern of isolation by distance (IBD) for early- and late-flowering landraces was investigated. Low IBD slopes were obtained for early flowering (*b*_E_ = -2.41E-05) and late-flowering (*b*_L_ = -9.89E-05) landraces (**Figure 5**). Similar results were found for logarithmic distances. This suggests no significant pattern of isolation-by-distance.

### *PgPHYC* and *PgMADS11* Allele Diversity

Landraces were genotyped for both *PgPHYC* and *PgMADS11* alleles to assess genetic diversity in relation to flowering time (**Table 2** and Supplementary Figures S4, S5). Only early landraces carried early flowering allele (G) at the *PgPHYC* locus leading to a significant difference in genotypes frequencies (*F*-test, *p* = 0.006). For *PgMADS11*, a significant difference in genotypes frequencies (*F*-test, *p* = 0.004) was observed with early flowering landraces having higher frequency of the allele (363 bp fragment). A significant correlation between allele G from *PgPHYC* with latitude (*p*-value = 0.0348) was observed. However, significance disappeared when taking into consideration genetic clustering (*q* ≤ 0.7). For *PgMADS11*, data reveal a significant correlation with longitude (*p*-value < 0.001) even when considering genetic clustering in the mode.

**Table 2:** Genotypes counts for improved varieties, early- and late-flowering landraces with *q*-values ≤ 0.7.

	*PgPHYC* alleles			*PgMADS11* alleles		
	C/C	C/G	G/G	363/363	363/387	387/387
Improved varieties	8	3	1	1	2	9
Early flowering landraces	239	21	0	18	65	185
Late-flowering landraces	79	0	0	2	16	62


## Discussion

### Large and Untapped Diversity in Senegalese Germplasm

In this study, a relatively high genetic diversity (*H*_Exp_: 0.516) of pearl millet germplasm of Senegal is reported. Previous studies have reported a higher (*H*_Exp_: 0.69) (Stich et al., 2010) and a lower (*H*_Exp_ = 0.49) genetic diversity in Niger pearl millet (Mariac et al., 2006a). These differences are due to the number of SSRs used. In the meanwhile, a similar number of alleles per locus (6 alleles per locus for Senegal vs. 6.2 alleles per locus for Niger) was observed. Comparing heterozygosity levels, observation showed a higher coefficient (*F*_IS_ = 0.30) in Niger germplasm than in Senegal germplasm (*F*_IS_ = 0.18). However, these results are consistent with data from others studies carried out on accessions across SSA regions and India (Oumar et al., 2008; Dussert et al., 2015; Hu et al., 2015). Pearl millet shows a high genetic diversity that can be explained by its strong outcrossing rate (75%) and the still on-going gene flow with its wild relative (Mariac et al., 2006a; Lewis, 2010). This high genetic diversity is in line with its high phenotyping diversity observed in Senegal (Sy et al., 2015) as in Western Africa (Pucher et al., 2015). Together, these findings highlight the untapped potential of Senegalese pearl millet germplasm for breeding.

Genetic proximity between early flowering varieties and improved varieties highlight a history of breeding programs and agricultural practices. In Senegal, few breeding programs have been undertaken on pearl millet but all focused on reducing the flowering cycle and were “population” varieties whose parental seeds were collected from local landraces. In addition, farmers still grow improved varieties jointly with landraces in their field, increasing gene flow and thus genetic proximity.

### Genetic Structure of Early- and Late-Flowering Landraces

Bayesian results clearly help identifying genetic structure associated with early- and late-flowering landraces. This genetic structure was partly explained by the geographic distribution of landraces as shown through the sPCA analysis, making it difficult to entirely disentangle spatial and genetic structure. More contrast in admixture patterns might have been obtained with a higher number of SSR markers. However, this was compensated by our sampling effort and we were able to reveal a clear structure in Senegal landraces where other studies failed with a higher number of markers (Hu et al., 2015).

At a local scale, more contrasted admixture patterns were observed. In Central Senegal, clear differentiation between early- and late-flowering landraces was found. In contrast, more admixed patterns were observed in the Southern Senegal. The results might be related to the agricultural practices of the farmers, such as spatial cropping and seed circulation (Mariac et al., 2006b; Lakis et al., 2012; Kouakou et al., 2013).

Our data indicated two distinct genetic clusters in Senegalese pearl millet germplasm. In addition, comparing early- and late-flowering landraces, we found a slight genetic differentiation between the two groups of 0.052, which is of the same magnitude (0.053) found in Niger (Lakis et al., 2012). A single domestication event led to early- and late-flowering landraces, partly explaining the limited genetic differentiation observed at a regional scale (Dussert et al., 2015).

### Flowering Traits Diversity

Photoperiod-sensitivity of pearl millet landraces and thus variation of flowering cycle constitute a key response for adaptation to future climate conditions (Sultan et al., 2013). Indeed, pearl millet landraces from Niger show reduced flowering cycle associated with drought episodes from 1976 to 2003 (Vigouroux et al., 2011). This reduction was correlated with changes in allele frequencies for *PgPHYC*. Correlation between allele frequencies and rainfall were also found with *PgMADS11* (Saïdou et al., 2009; Mariac et al., 2011). Our data showed significant genetic differences for both genes with early flowering landraces enriched in precocity alleles.

Flowering time has been correlated with latitude, early flowering landraces being grown in northern latitude where environmental conditions are more arid (Haussmann et al., 2006; Pucher et al., 2015). In our study, the latitude effect was confounded with spatial genetic structure for *PgPHYC*. On the other hand, a correlation with longitude was found for *PgMADS11*. Further investigation would be needed to fully address this correlation. In any case, the use of flowering genes *PgPHYC* and *PgMADS11* in marker assisted-selection programs presents some interest.

### Challenges for Adaptation of Pearl Millet and Breeding Strategies

Sub-Saharan Africa recorded long dry spells in the 1970s and 1980s that led to breeding for short cycles improved varieties. Indeed, pearl millet breeding programs were predominantly built on restricted genetic resources of early flowering landraces. Strong differentiation between early- and late-flowering landraces from Senegal suggests the existence of an important gene pool that has not been exploited yet. The high genetic diversity could explain the wider range of pearl millet adaptation to dry areas and this potential may further contribute to breeding programs in response to the specific needs or target areas (Kouressy et al., 2004). For instance, a key strategy to cope against climate changes within SSA agrosystems is to tap into diversity of flowering time (Haussmann et al., 2012; Sultan et al., 2013) and resilience (Prieto et al., 2015).

Analyses of allelic variation of *PgPHYC* and *PgMADS11* indicate fine-scale genetic difference (SNP and indel, respectively) among individuals and/or genotypes. Knowing that responses to photoperiod and rainfall were genetically associated with both genes, implication could be their use to detect/track climate adaptive changes to environment variations. For example, earliness of flowering and latitude correlation observed in early landraces support assumption that a direct effect of selection for that trait which is associated with climate variations such as photoperiod and rainfall. Both traits are key targets in selection for millet genotypes to be cultivated in rainfed areas.

## Conclusion

The genetic diversity and population structure of Senegalese pearl millet landraces were assessed using a large panel of accessions and a limited number of SSRs markers. Results highlight a high genetic diversity and an untapped potential of the germplasm. However, two clusters were clearly distinguished as revealed by differentiation between early- and late-flowering landraces. Further, genetic difference and allelic co-variation in flowering genes *PgPHYC* and *PgMADS11* were found among individuals. These findings give new insights into Senegalese pearl millet germplasm and are promising for developing new cultivars and heterotic groups that can be used to breed synthetic and hybrid varieties with higher degrees of heterozygosity in order to intensify yield production under harsh semiarids environments.

## Author Contributions

NK, AF, DD, MG, YV, and AB designed the study. OD, AF, MG, MP, HT, BD, and OS collected samples. OD, MC, MP, and LZ performed DNA extraction, PCR and sequencing. AB, CB-S, and OD performed the genetic analyses. OD, AB, CB-S, DD, and NK drafted the manuscript. All authors contributed to the final version.

## Conflict of Interest Statement

The authors declare that the research was conducted in the absence of any commercial or financial relationships that could be construed as a potential conflict of interest.
