# Supplementary material for: New Genetic Insights into Pearl Millet Diversity As Revealed by Characterization of Early- and Late-Flowering Landraces from Senegal
Source: Front Plant Sci. 2017 May 17;8:818. doi: 10.3389/fpls.2017.00818 (PMC5434141; doi:10.3389/fpls.2017.00818)
Supplement: Supplementary file 2 [file Data_Sheet_2.PDF]

Supplemental figures

Diack et al. New genetic insights of pearl millet diversity

A)

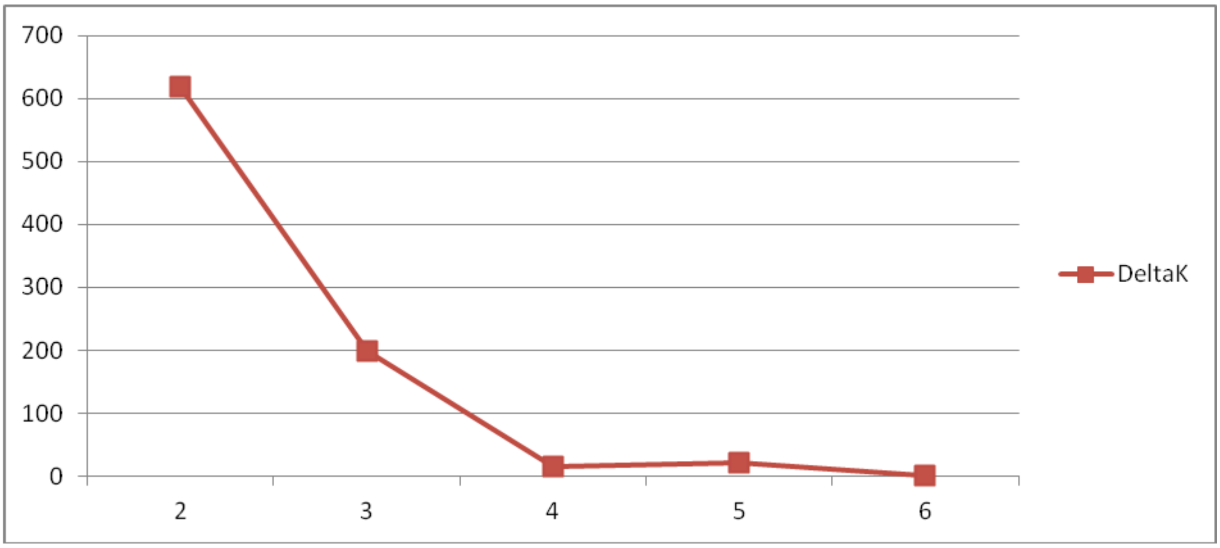

B)

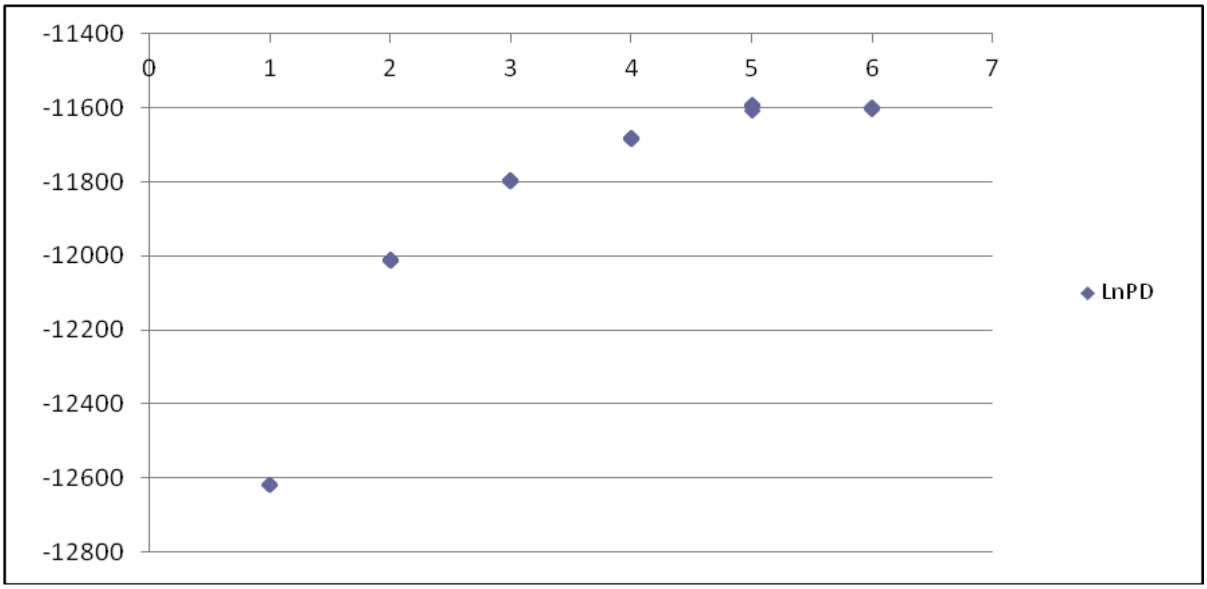

Figure S1. *D.ΔK* criterion according to the calculation method by Evanno et al. (2005) (A) and LnPD regression (for K=1 to K= 6 (K number of groups) (B). These results were obtained for 404 accessions analysed with 12 SSR markers. Higher D.ΔK indicates higher likelihood of genetic groups. Here, we observed a higher likelihood for two genetic groups (K=2). Inflection point and low variances of LnPD indicates higher likelihood of number of genetic clusters. Here, we observed a higher likelihood for two genetic groups (K=2), which confirms the observation found with the D.ΔK criterion.

Figures S2: SSR profiles for makers Psmp2202 among 35 accessions

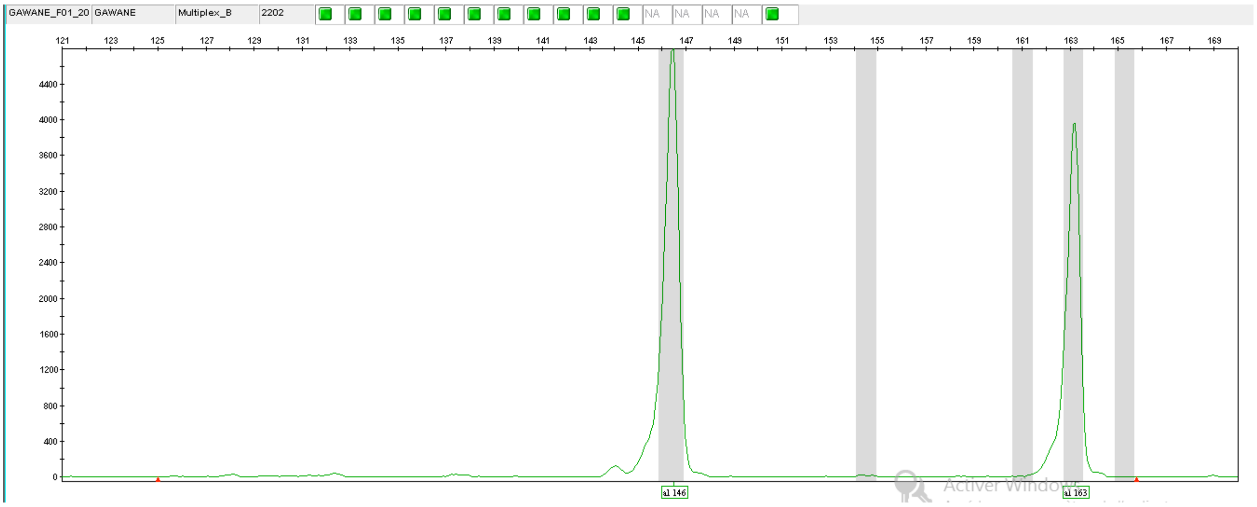

1: Microsatellite marker *Psmp2202* profile on sample GAWANE

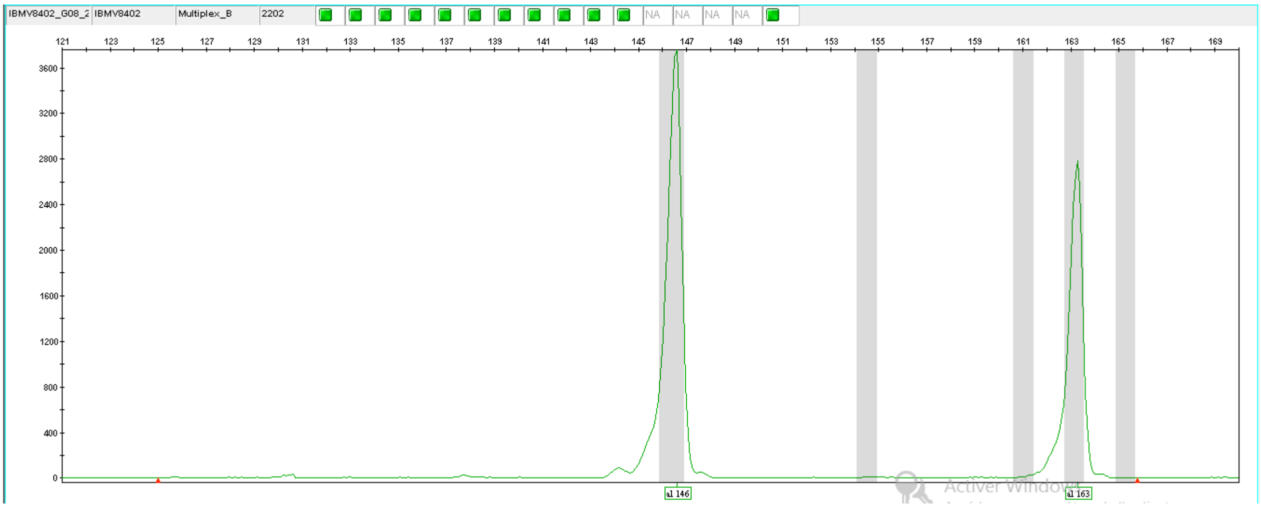

2: Microsatellite marker *Psmp2202* profile on sample IBMV_8402

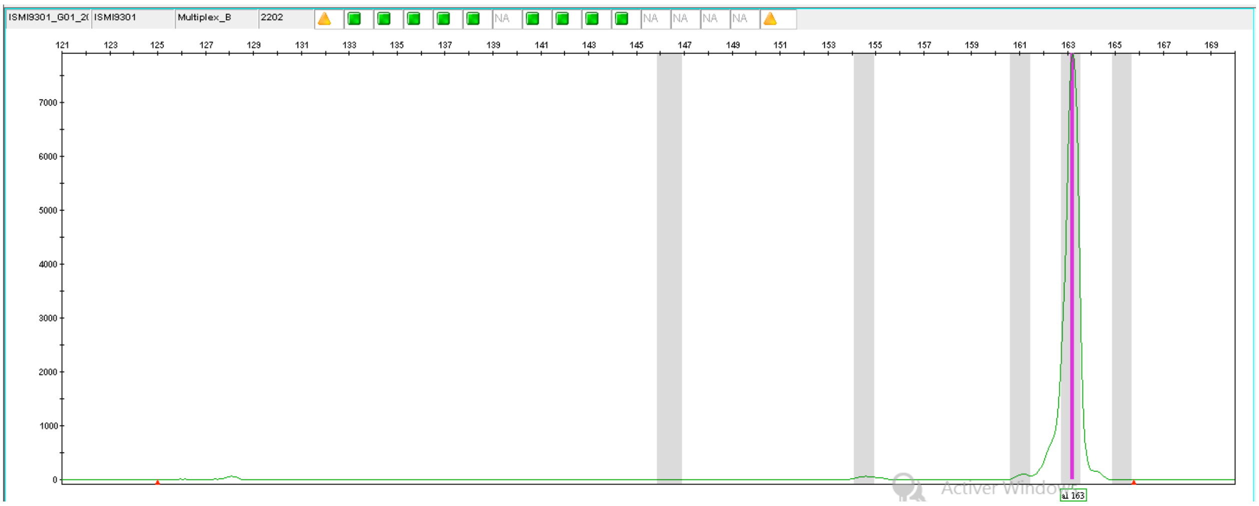

3 : Microsatellite marker *Psmp2202* profile on sample ISMI_9301

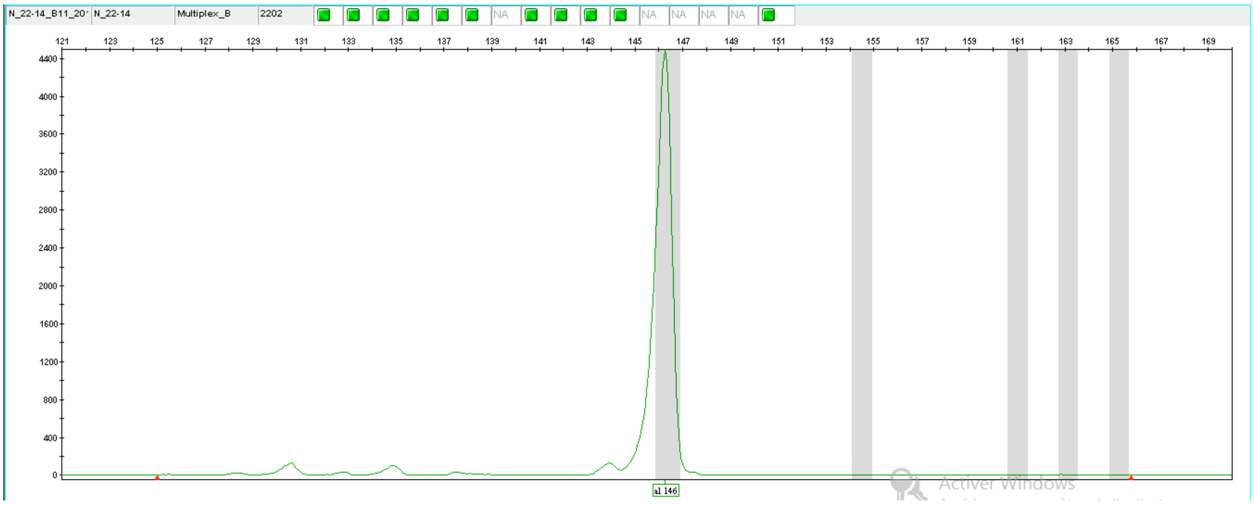

<u>4:</u> Microsatellite marker *Psmp2202* profile on sample N_22-14

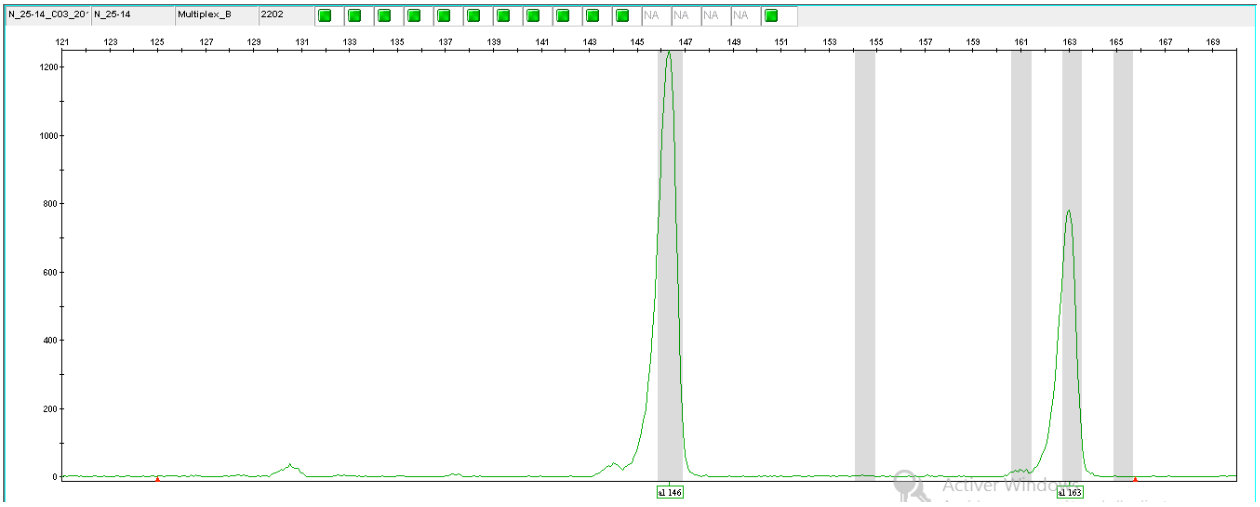

<u>5:</u> Microsatellite marker *Psmp2202* profile on sample N_25-14

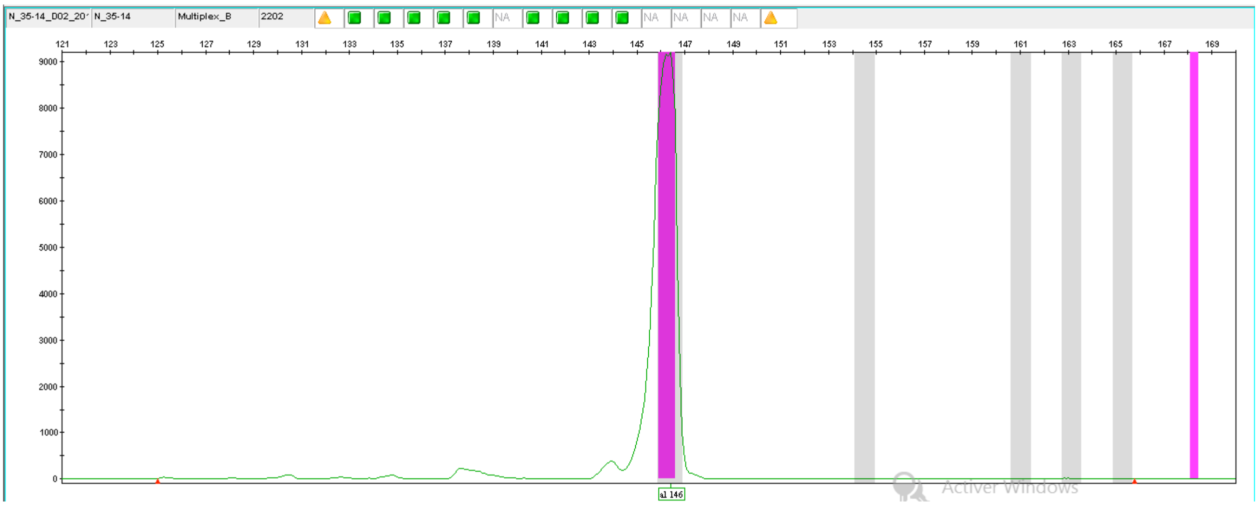

<u>6</u> : Microsatellite marker *Psmp2202* profile on sample N_35-14

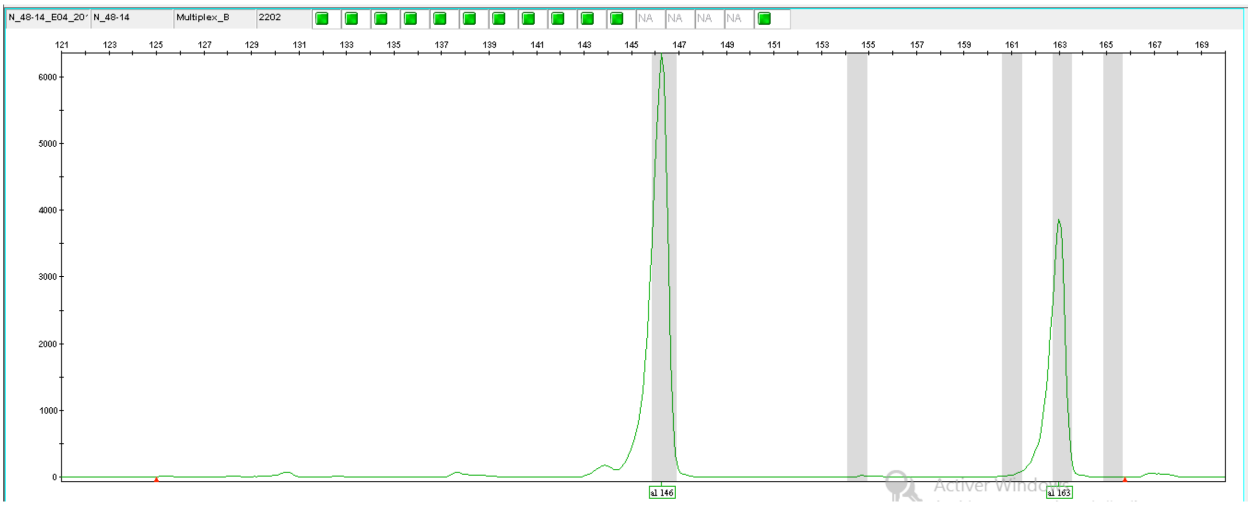

7: Microsatellite marker *Psmp2202* profile on sample N_48-14

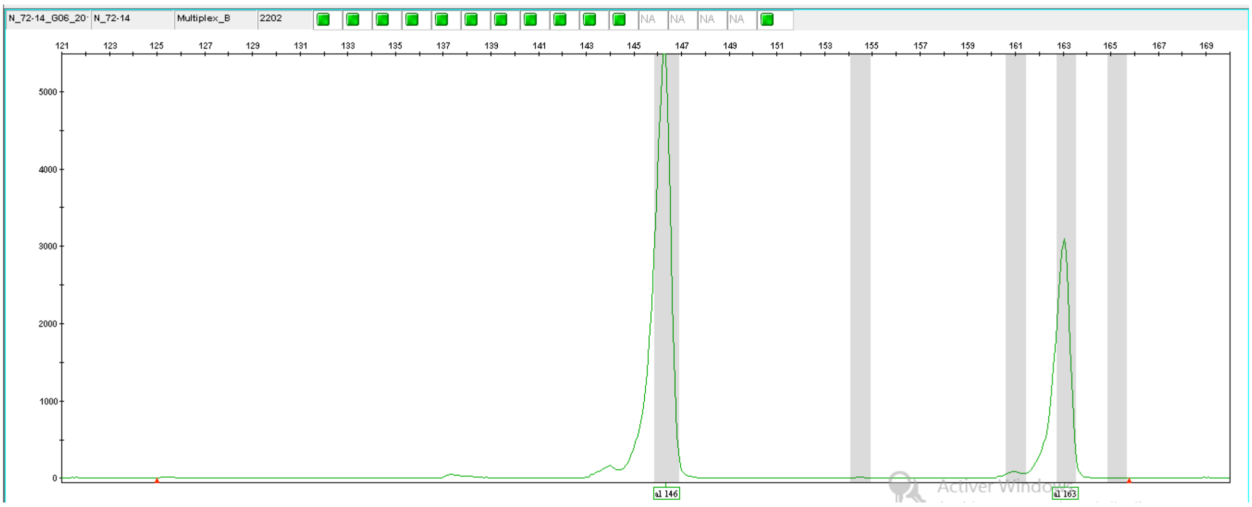

8: Microsatellite marker *Psmp2202* profile on sample N_72-14

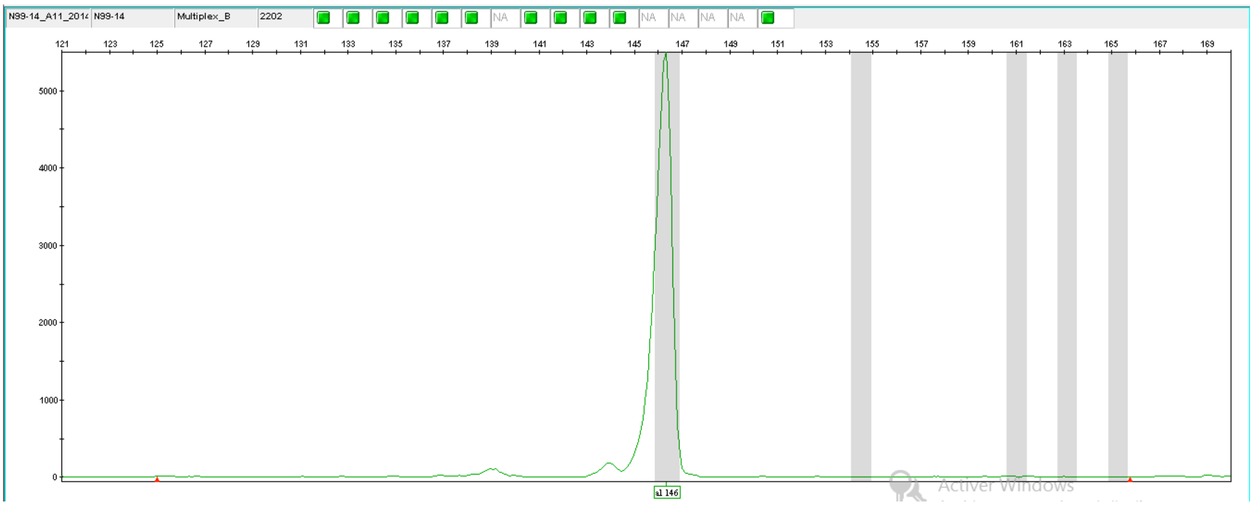

9 : Microsatellite marker *Psmp2202* profile on sample N_99-14

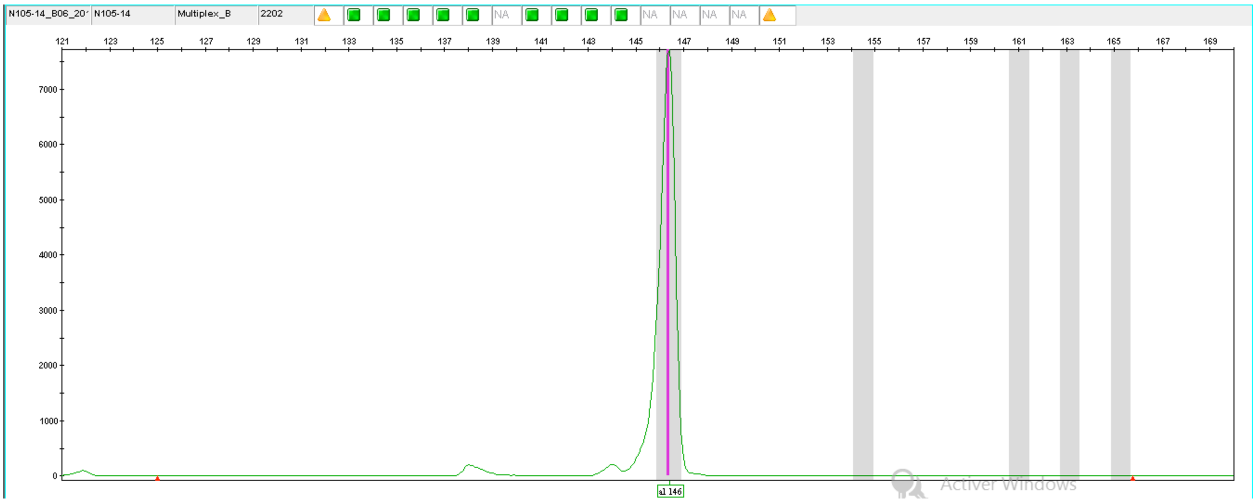

10: Microsatellite marker *Psmp2202* profile on sample N_105-14

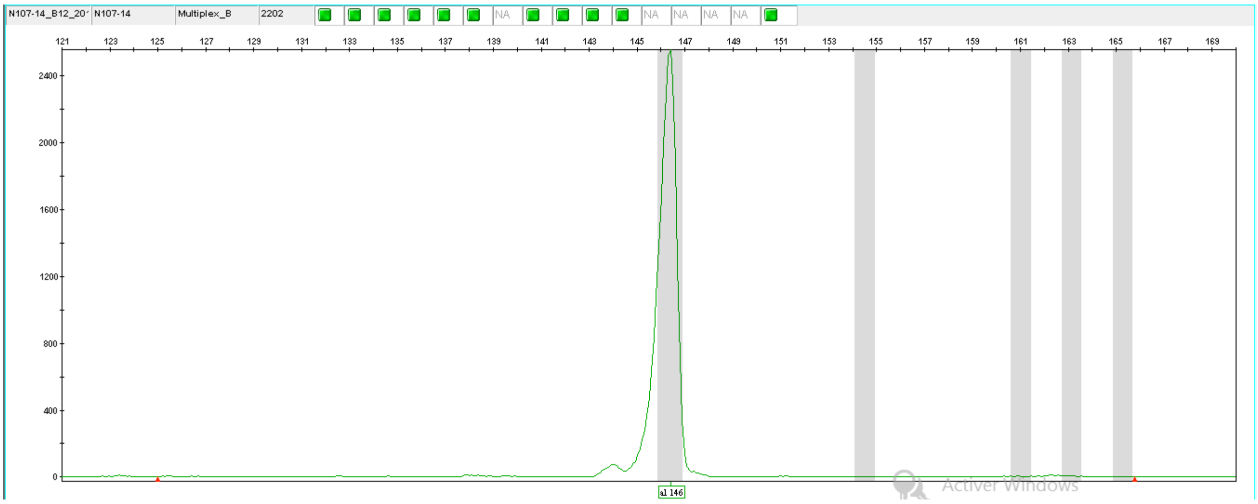

11: Microsatellite marker *Psmp2202* profile on sample N_107-14

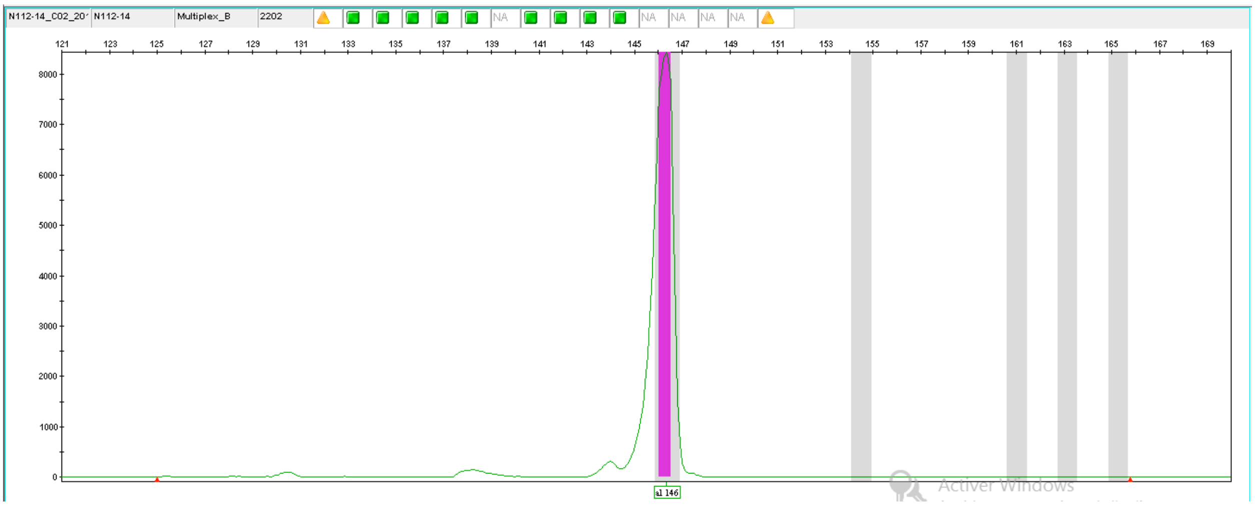

12 : Microsatellite marker *Psmp2202* profile on sample N_112-14

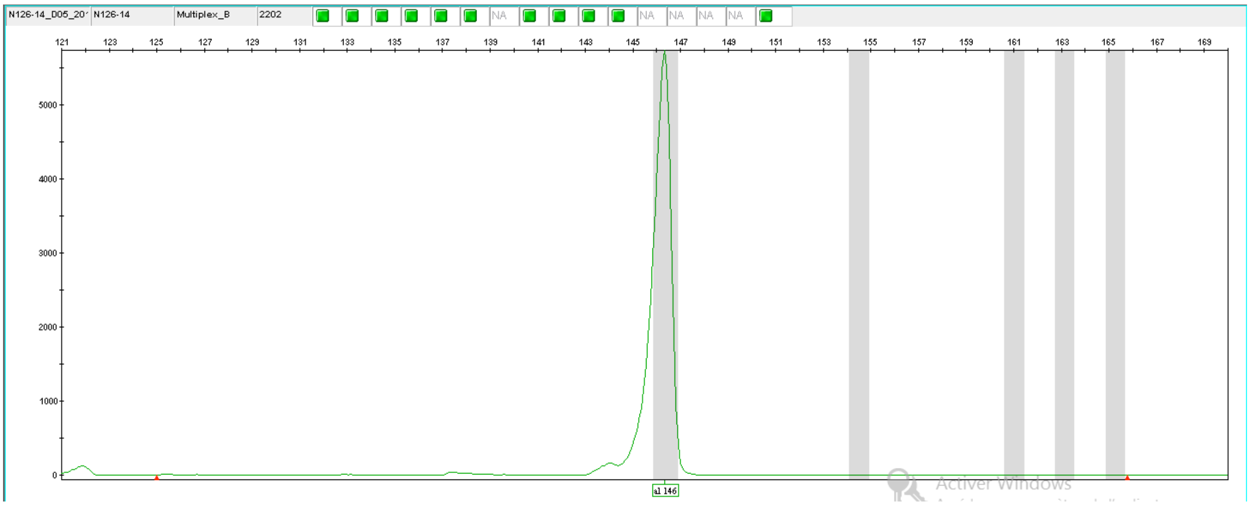

13: Microsatellite marker *Psmp2202* profile on sample N_126-14

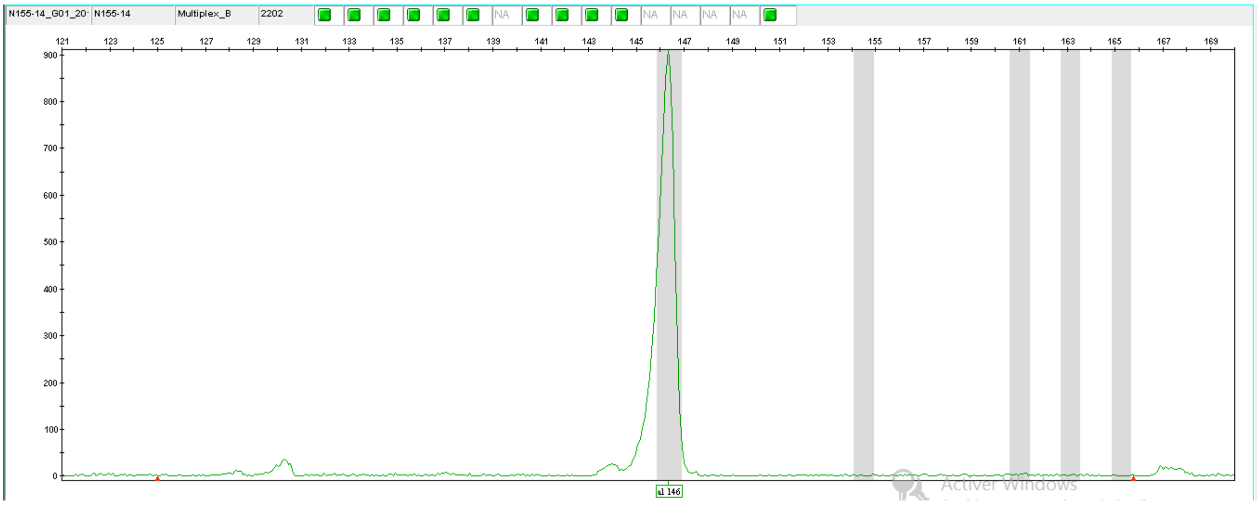

14: Microsatellite marker *Psmp2202* profile on sample N_155-14

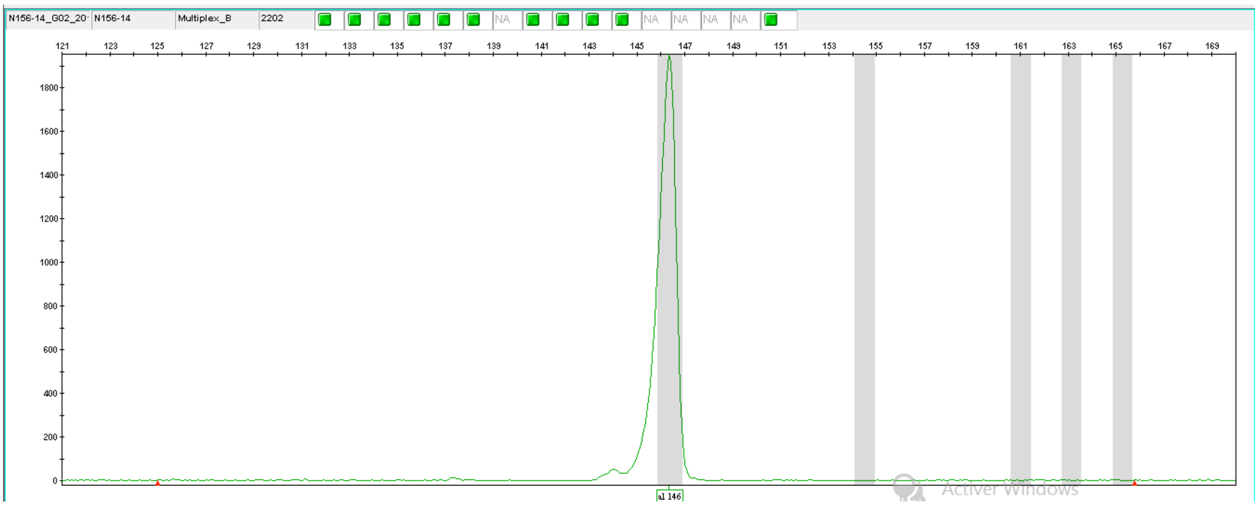

15: Microsatellite marker *Psmp2202* profile on sample N_156-14

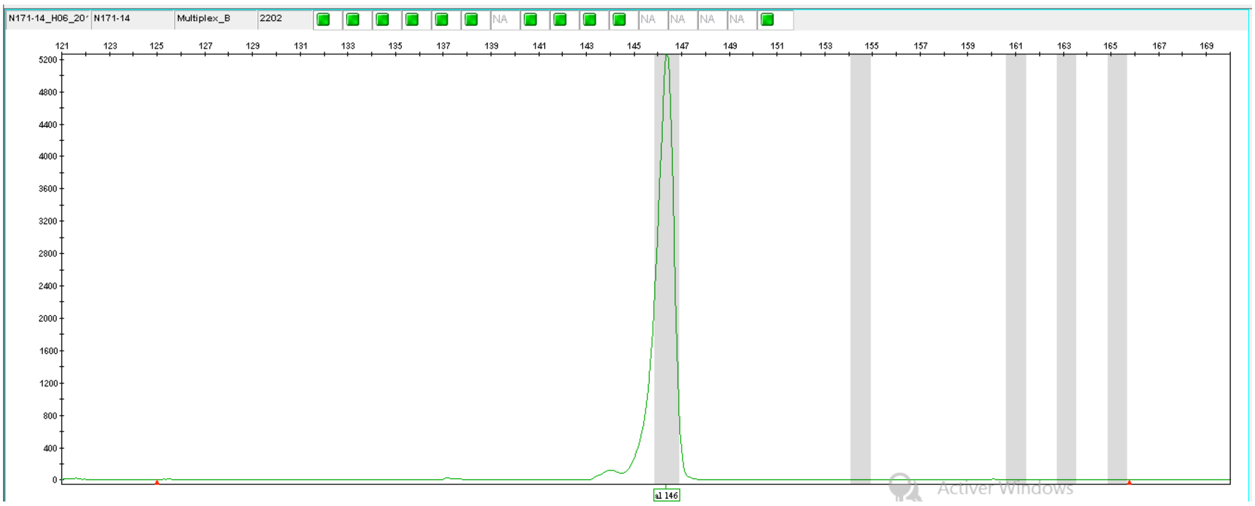

16: Microsatellite marker *Psmp2202* profile on sample N_171-14

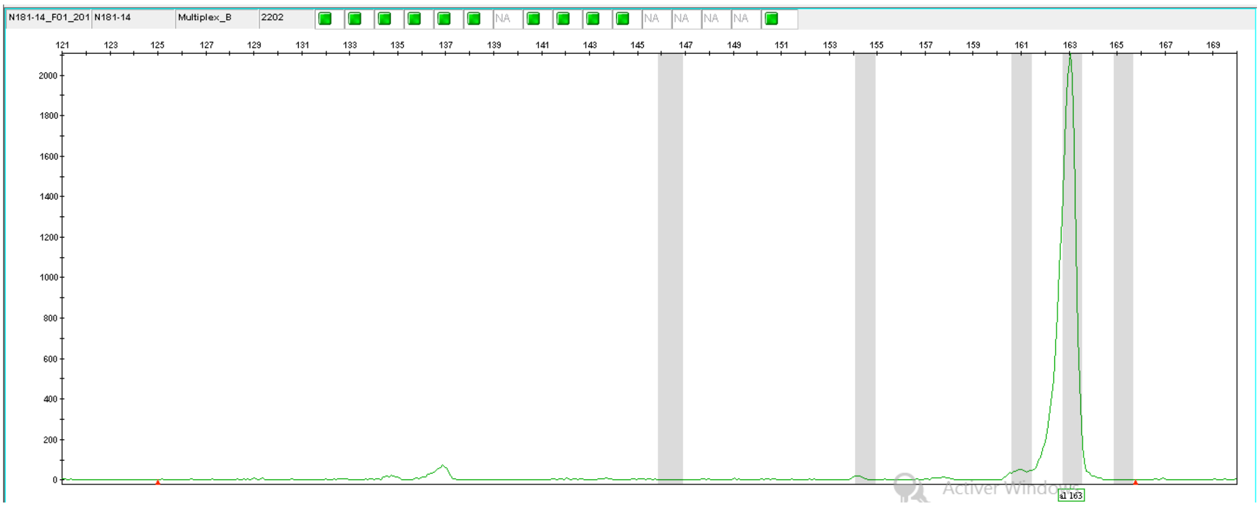

17: Microsatellite marker *Psmp2202* profile on sample N_181-14

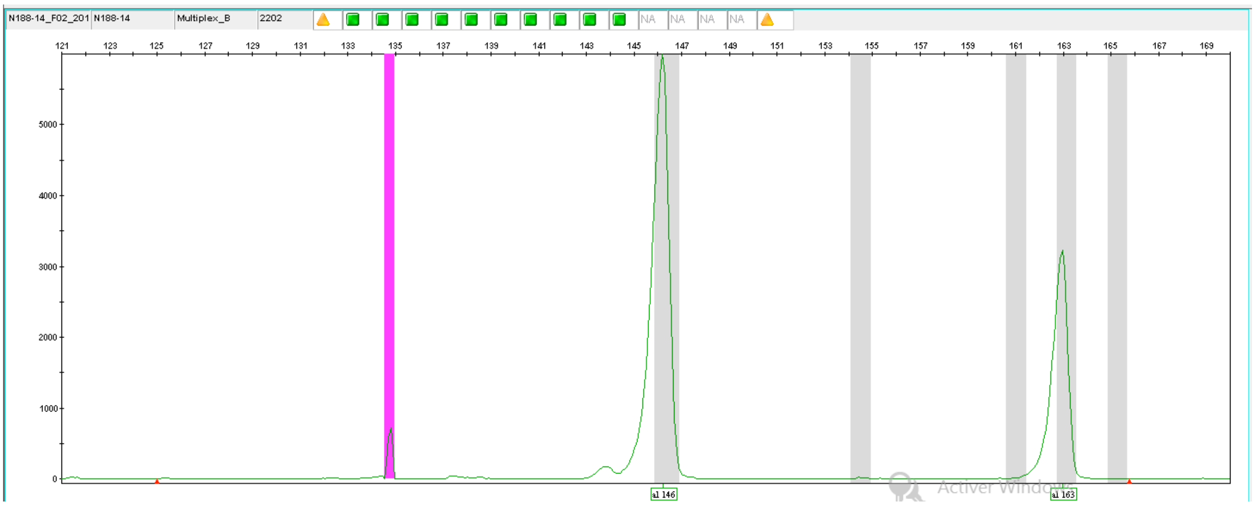

18: Microsatellite marker *Psmp2202* profile on sample N_188-14

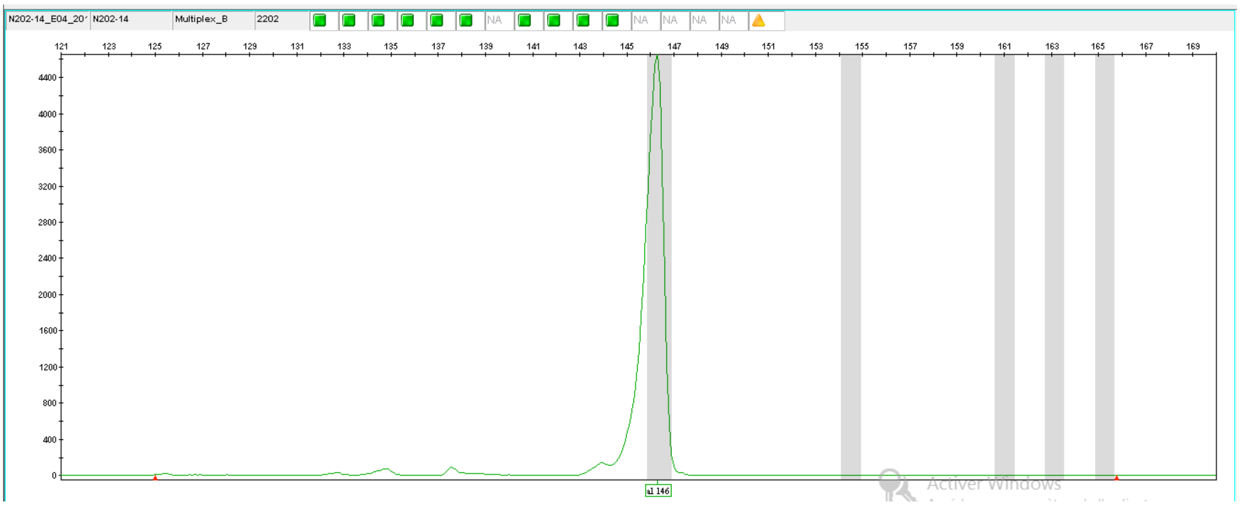

19: Microsatellite marker *Psmp2202* profile on sample N_202-14

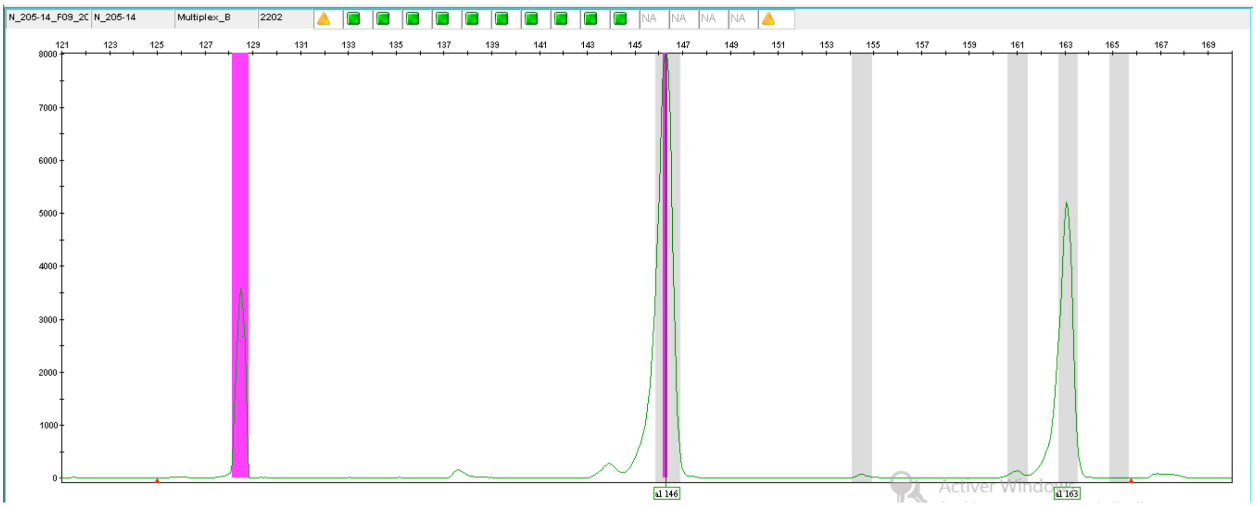

20: Microsatellite marker *Psmp2202* profile on sample N_205-14

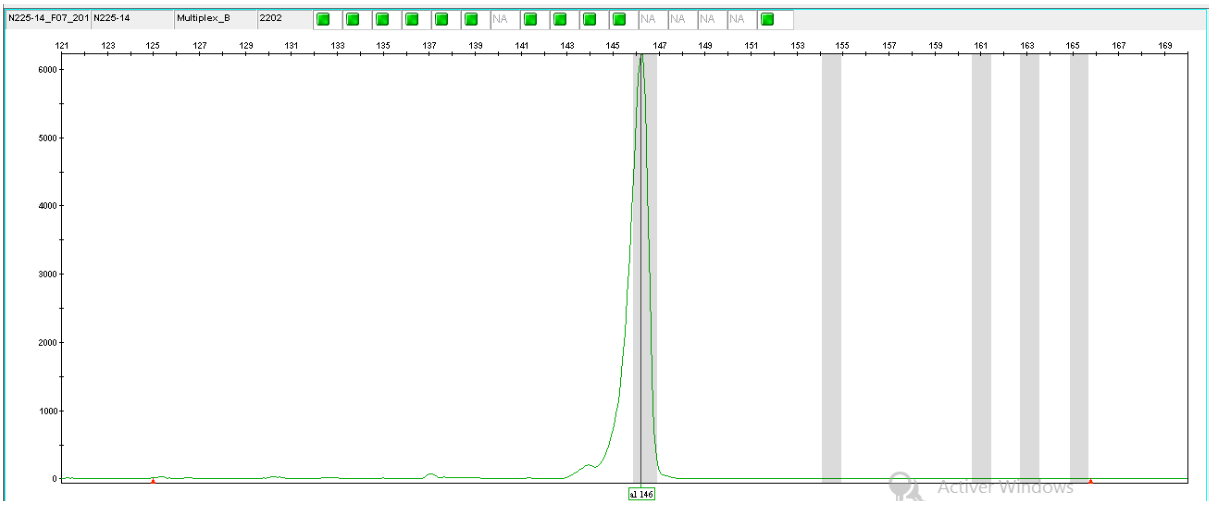

21: Microsatellite marker *Psmp2202* profile on sample N_225-14

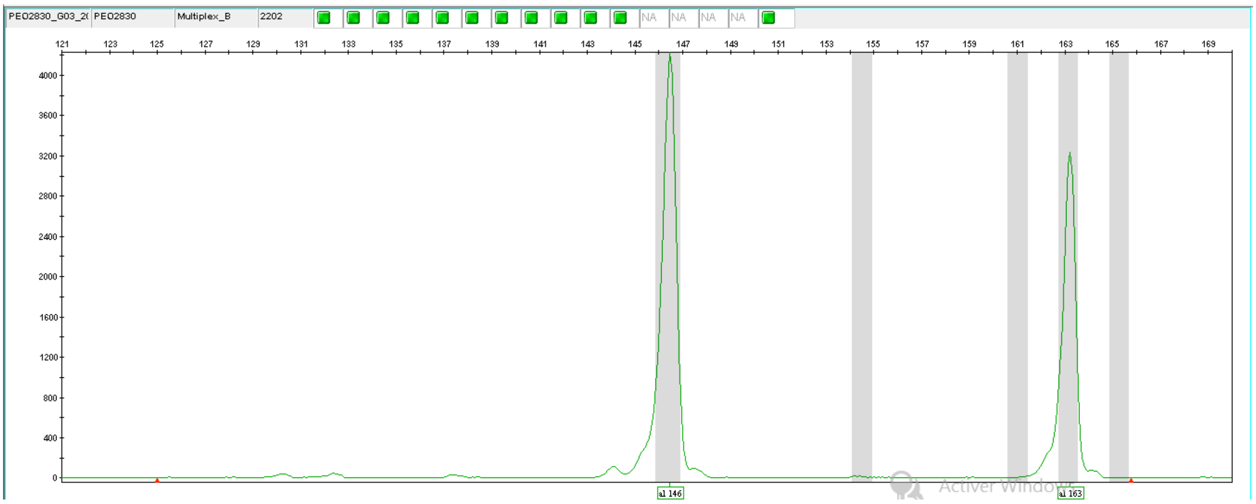

22: Microsatellite marker *Psmp2202* profile on sample PE0_2830

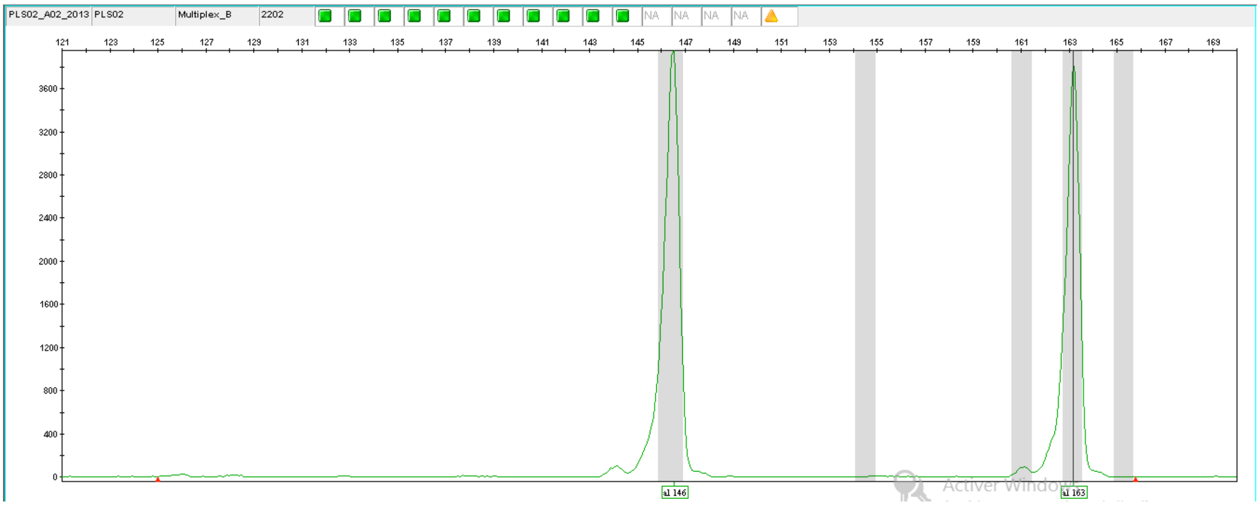

23: Microsatellite marker *Psmp2202* profile on sample PLS_02

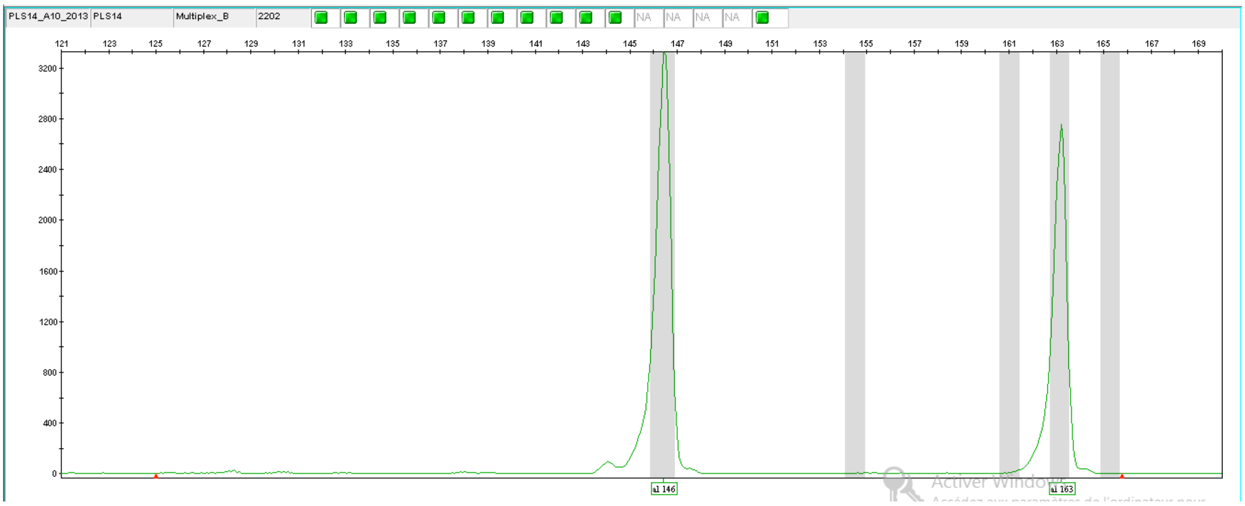

24: Microsatellite marker *Psmp2202* profile on sample PLS_14

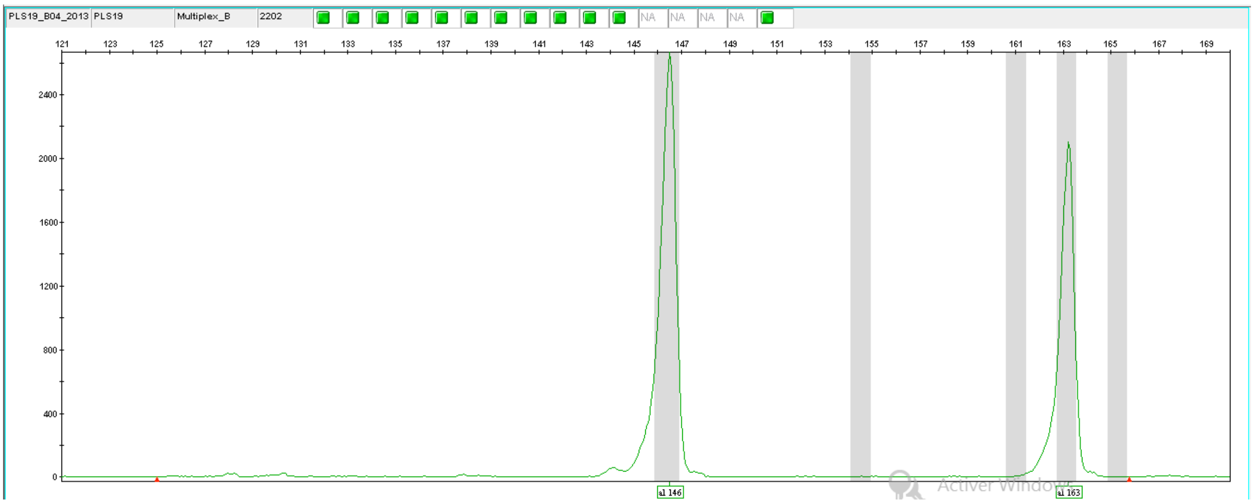

25: Microsatellite marker *Psmp2202* profile on sample PLS_19

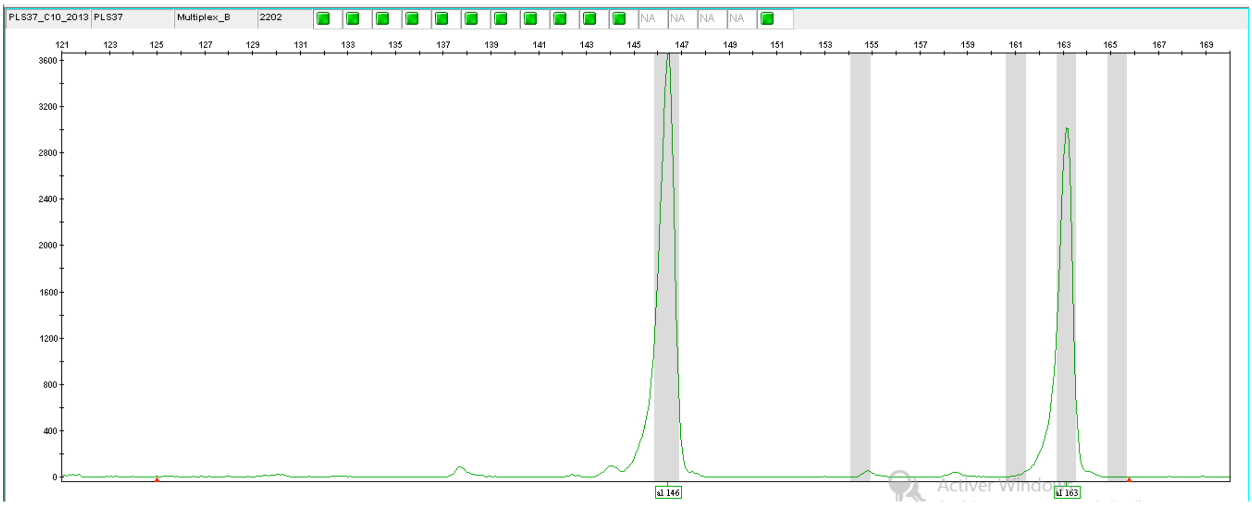

26: Microsatellite marker *Psmp2202* profile on sample PLS_37

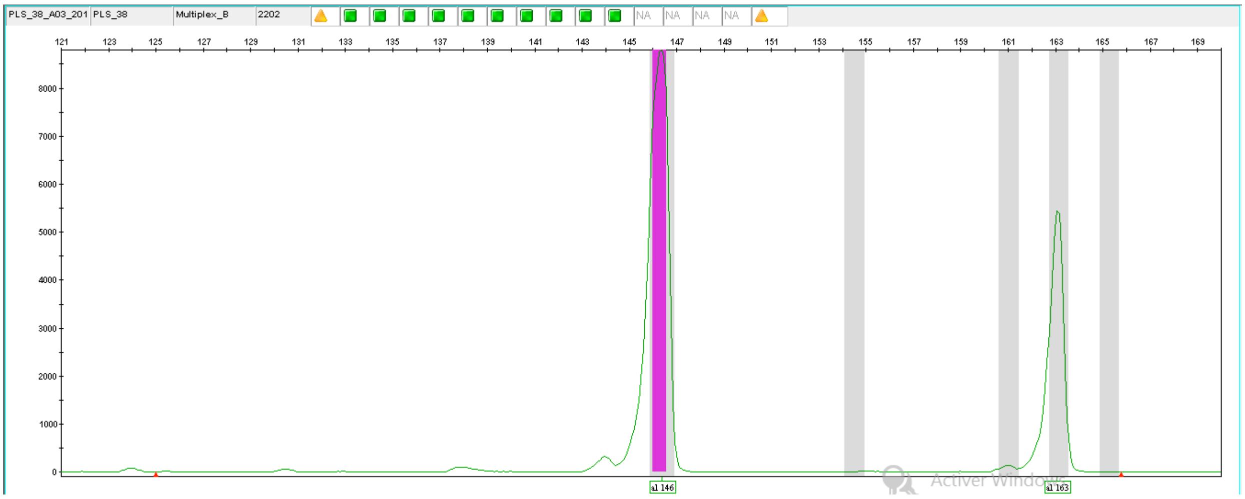

27: Microsatellite marker *Psmp2202* profile on sample PLS_38

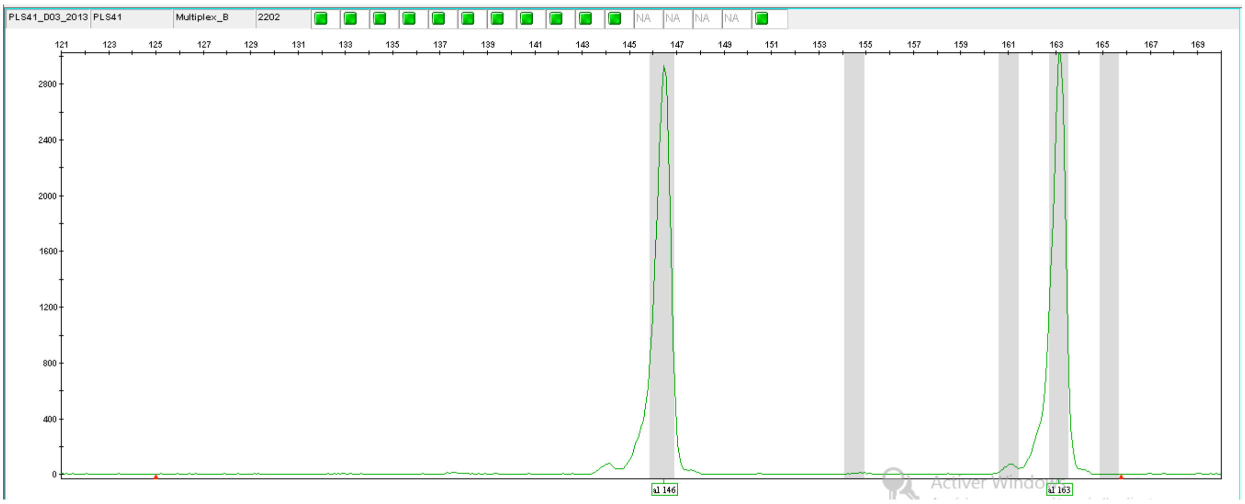

28: Microsatellite marker *Psmp2202* profile on sample PLS_41

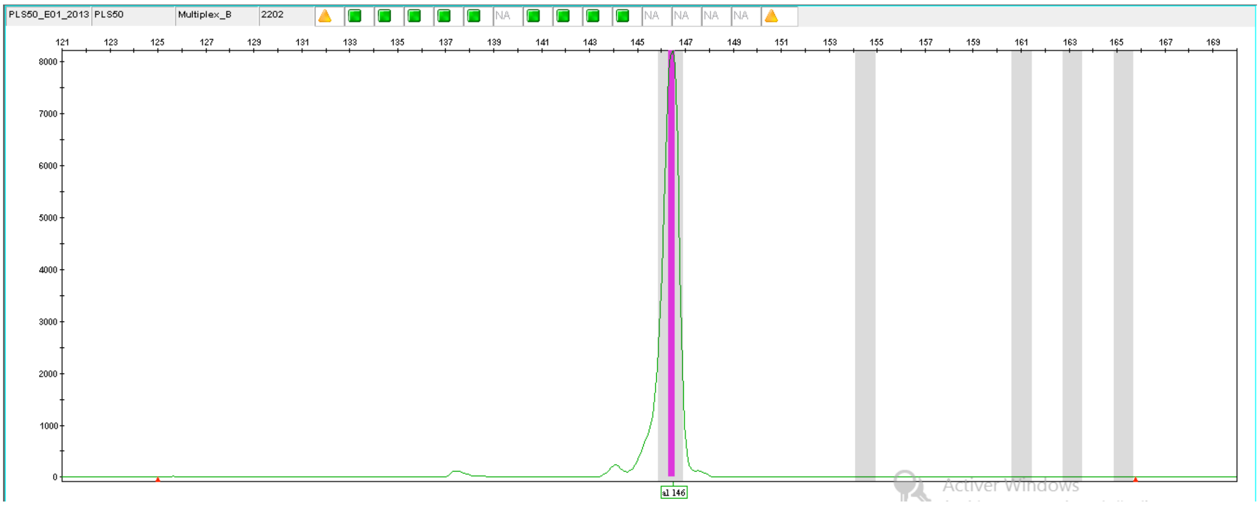

29: Microsatellite marker *Psmp2202* profile on sample PLS_50

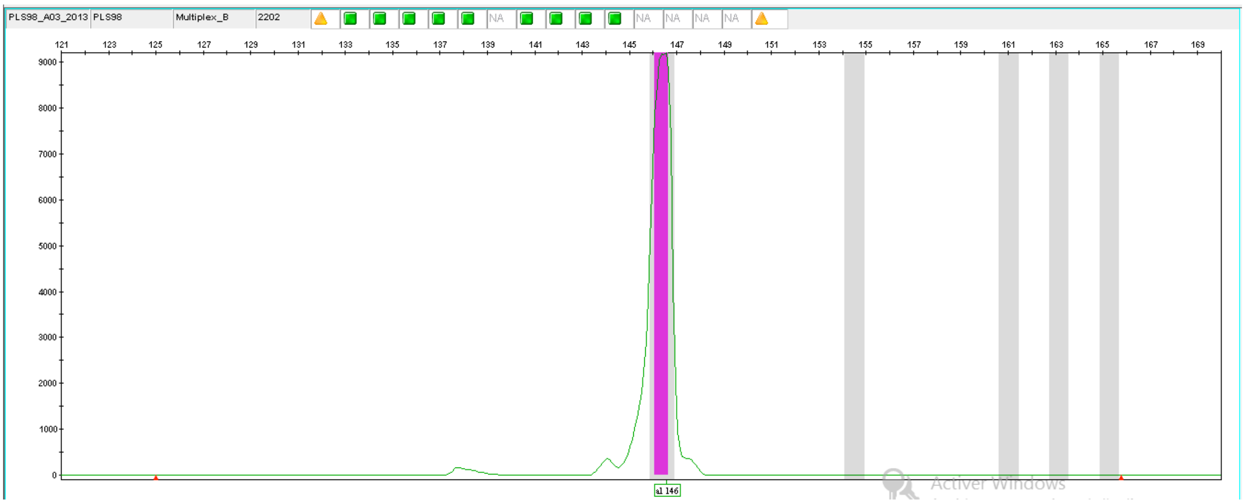

30: Microsatellite marker *Psmp2202* profile on sample PLS_98

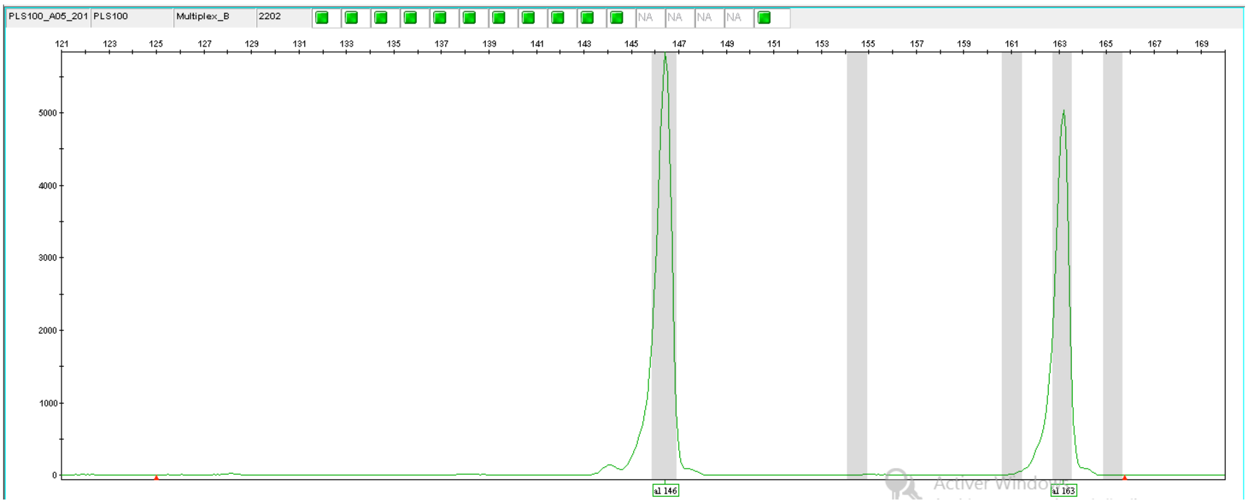

31: Microsatellite marker *Psmp2202* profile on sample PLS_100

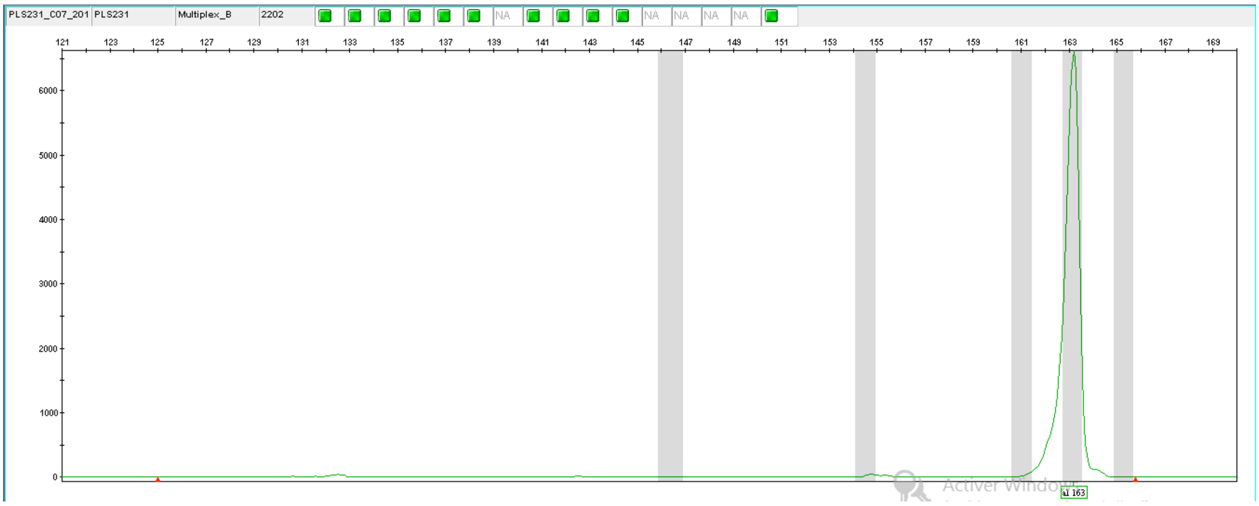

32: Microsatellite marker *Psmp2202* profile on sample PLS_231

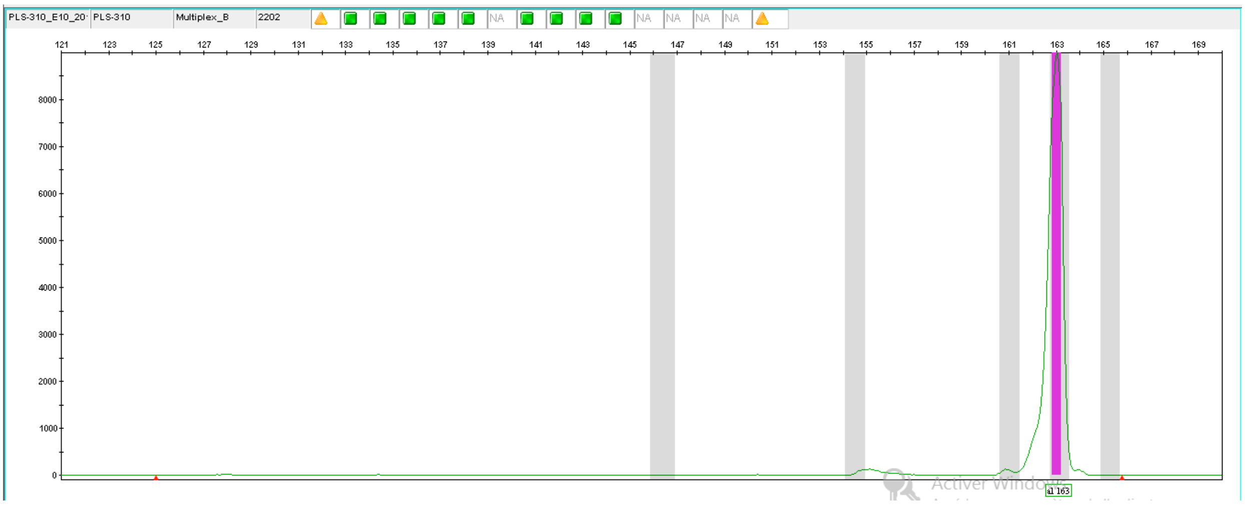

33: Microsatellite marker *Psmp2202* profile on sample PLS_310

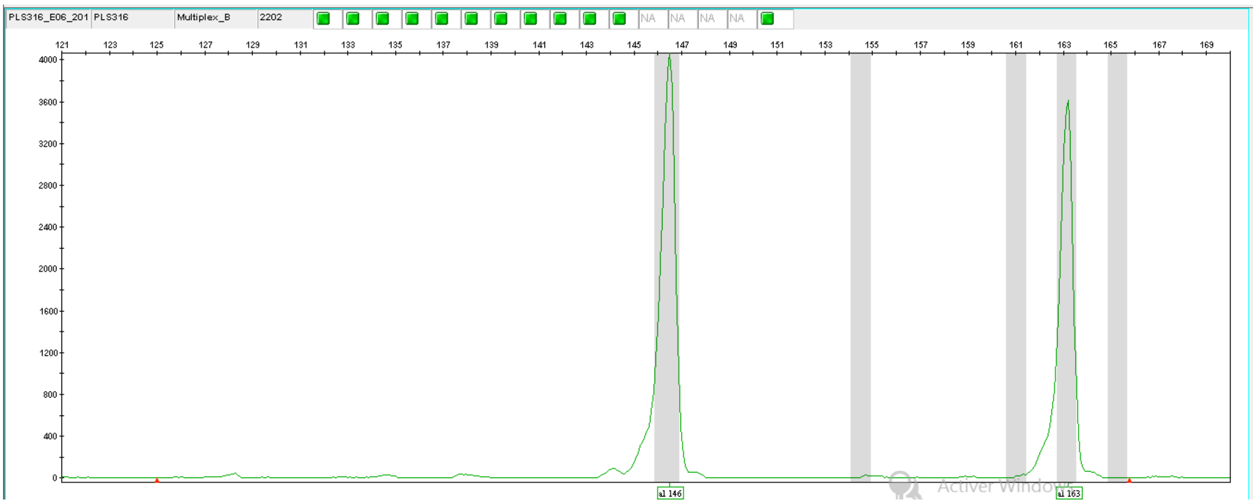

34: Microsatellite marker *Psmp2202* profile on sample PLS_316

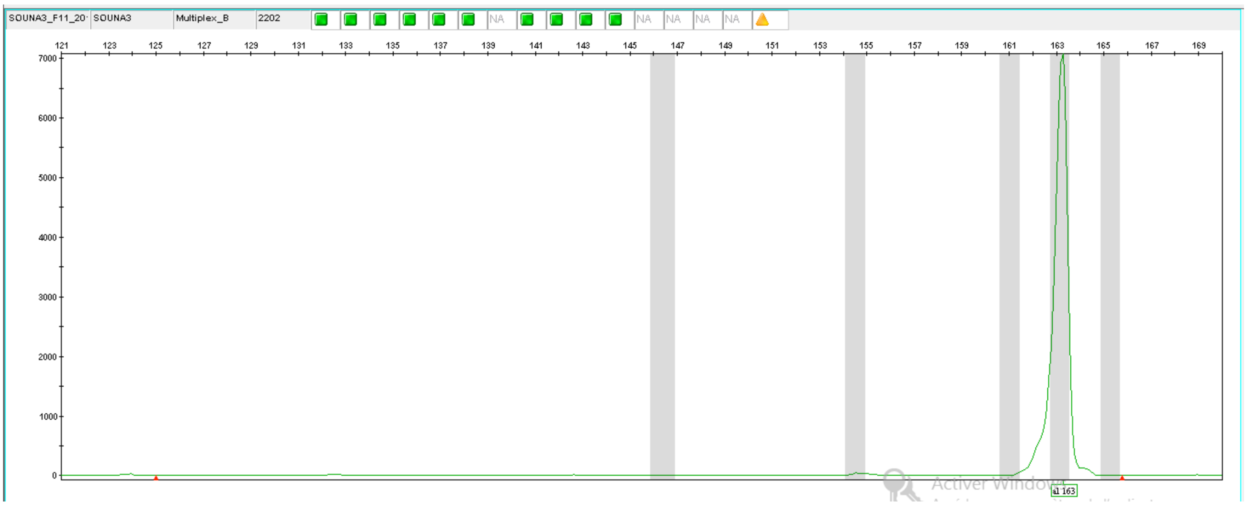

35: Microsatellite marker *Psmp2202* profile on sample SOUNA_3

Figures S3: SSR profiles for makers *PGIRD25* among 35 accessions

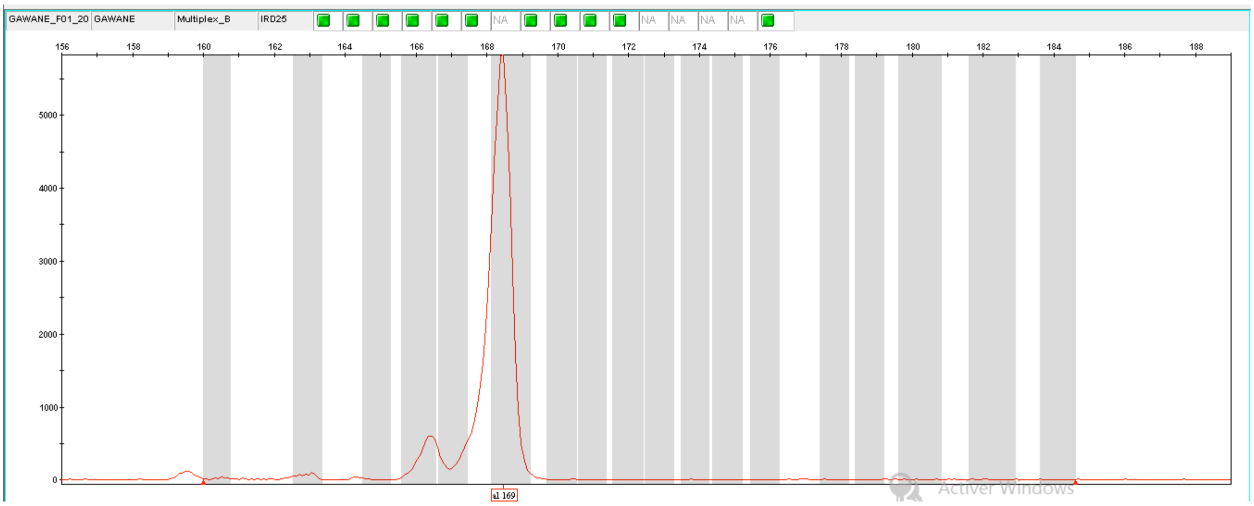

1: Microsatellite marker *PGIRD25* profile on sample GAWANE

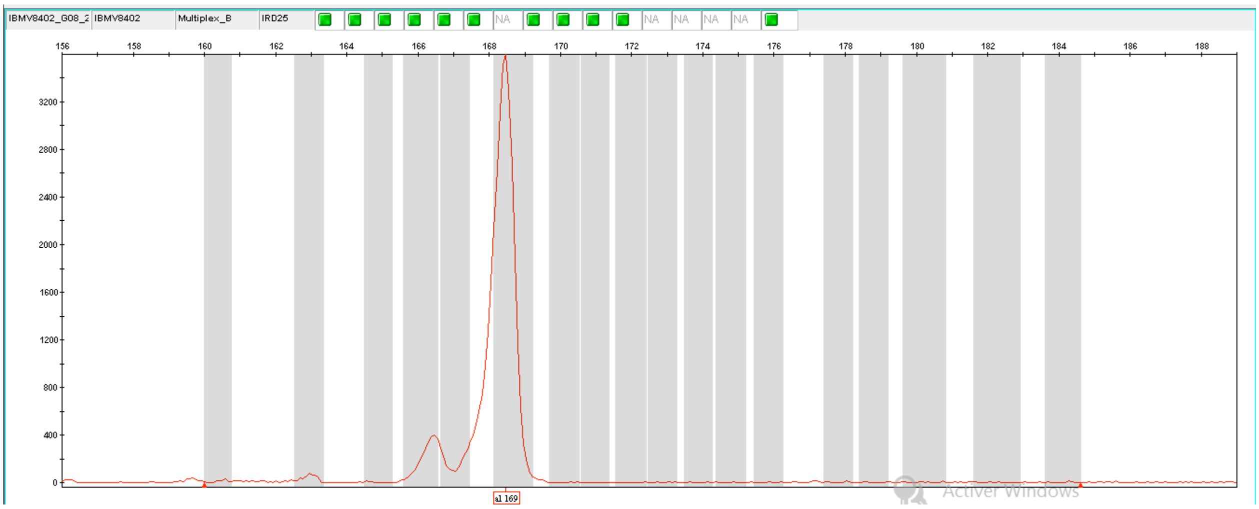

2: Microsatellite marker *PGIRD25* profile on sample IBMV_8402

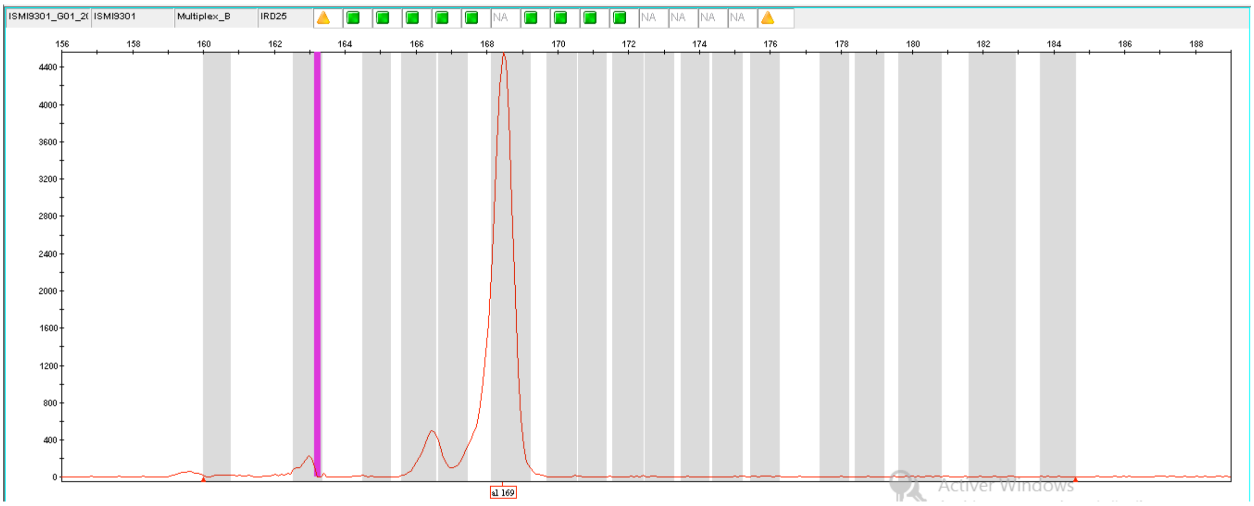

3 : Microsatellite marker *PGIRD25* profile on sample ISMI_9301

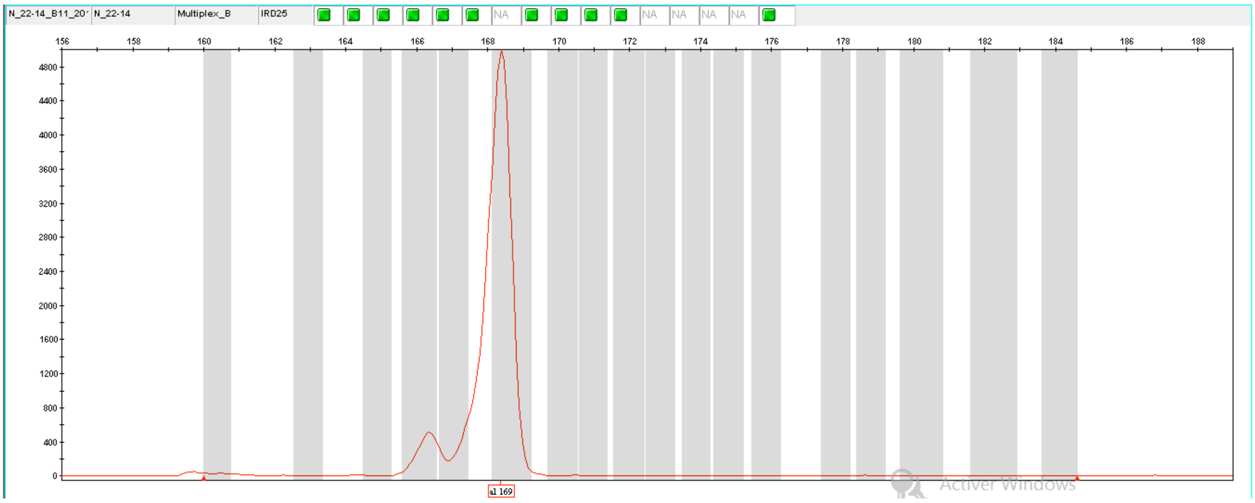

4: Microsatellite marker *PGIRD25* profile on sample N_22-14

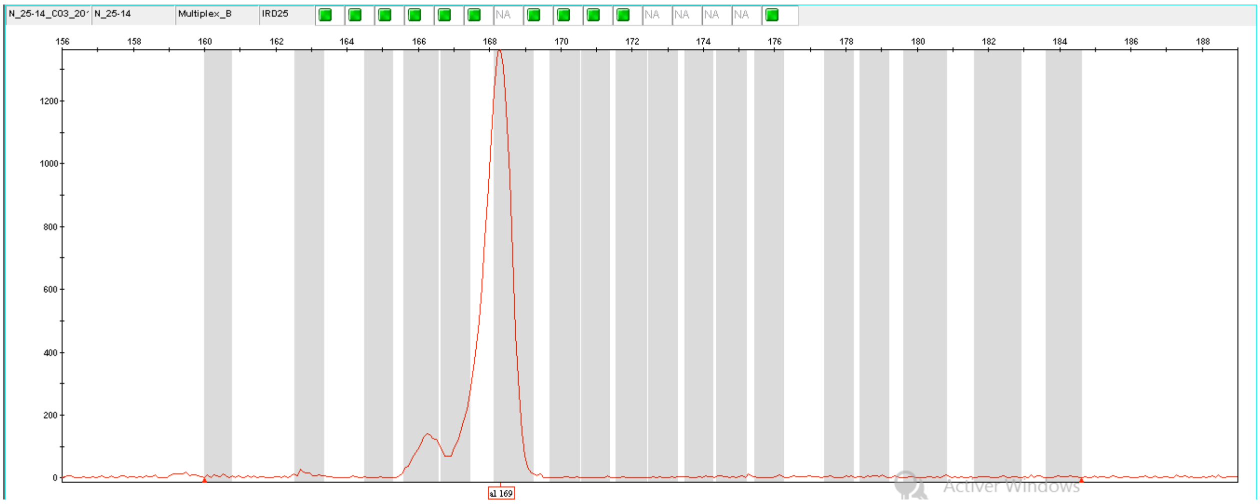

5: Microsatellite marker *PGIRD25* profile on sample N_25-14

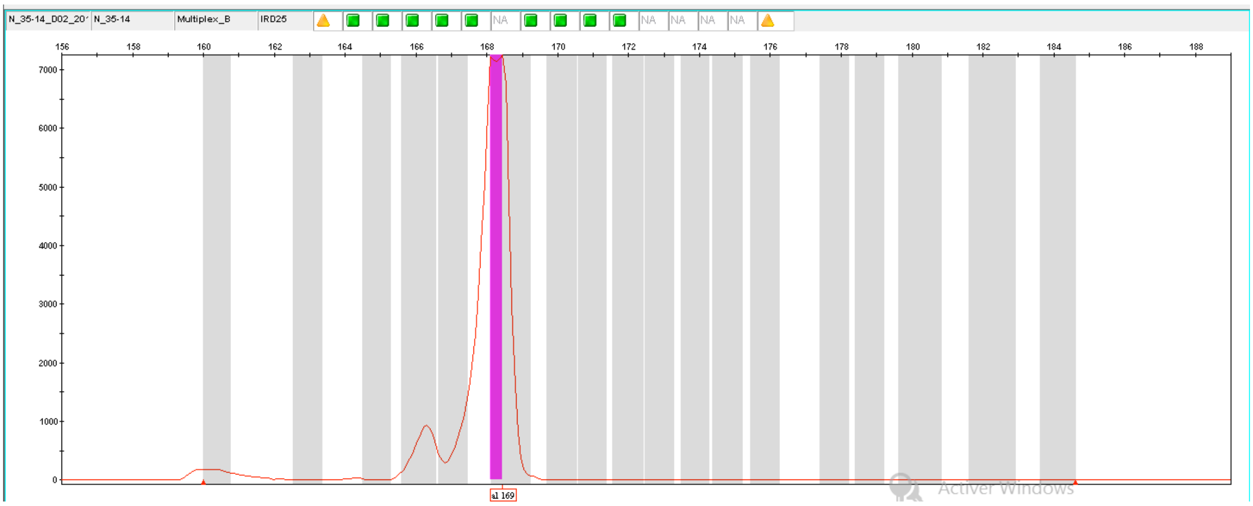

6 : Microsatellite marker *PGIRD25* profile on sample N_35-14

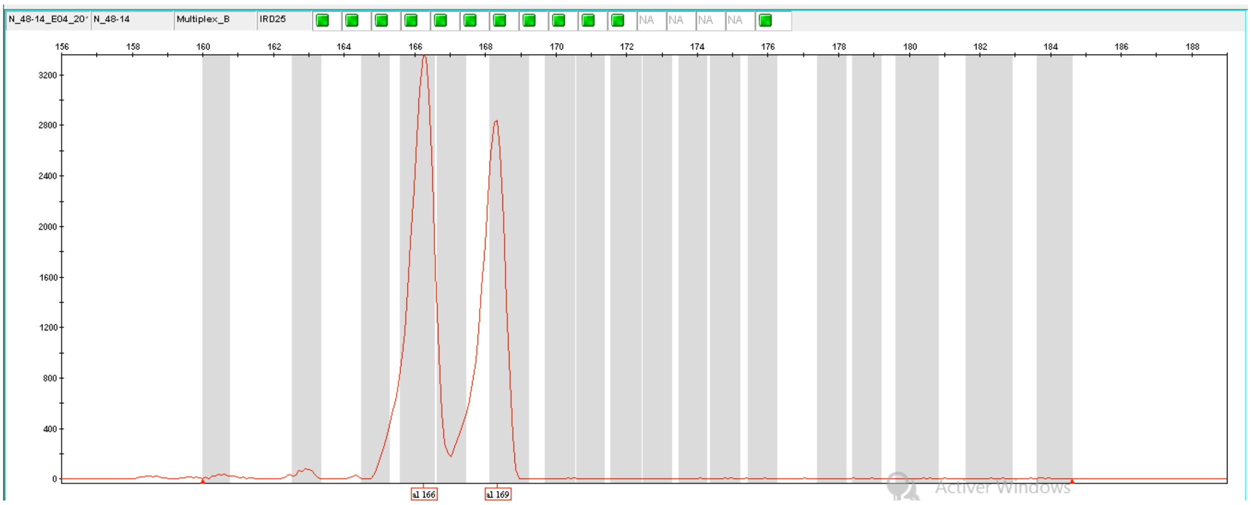

7: Microsatellite marker *PGIRD25* profile on sample N_48-14

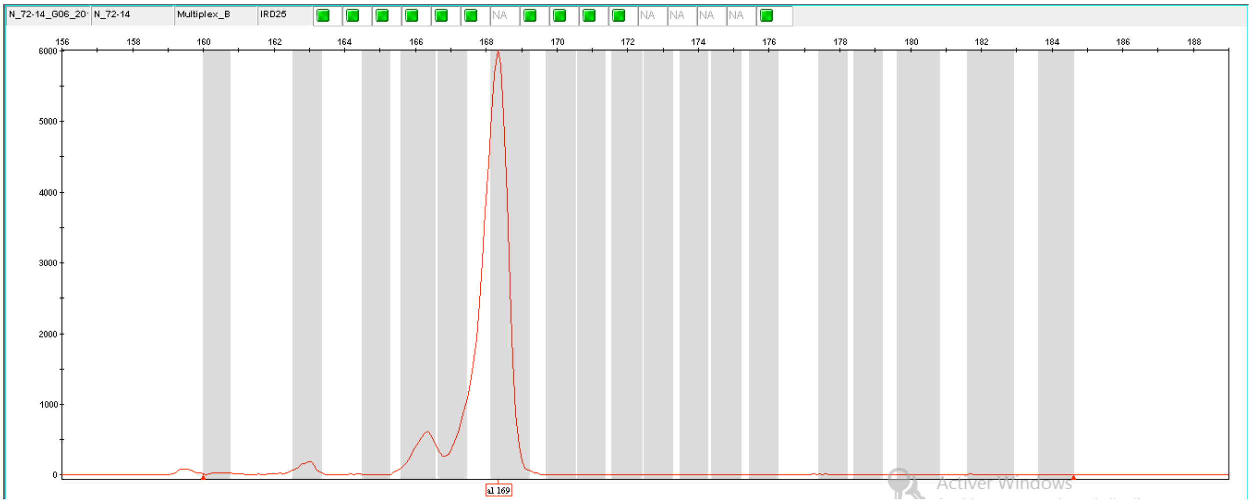

8: Microsatellite marker *PGIRD25* profile on sample N_72-14

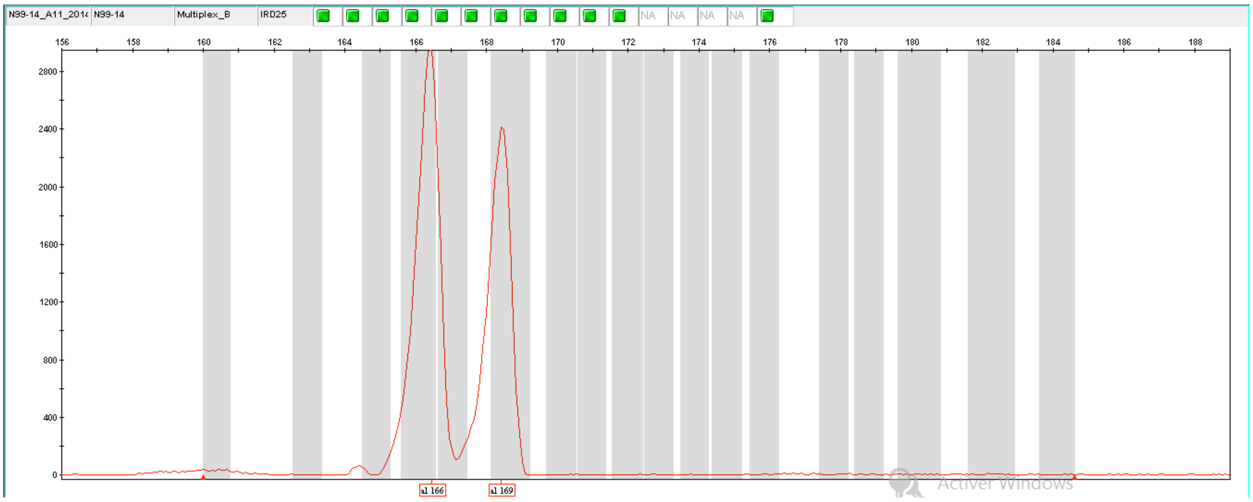

9 : Microsatellite marker *PGIRD25* profile on sample N_99-14

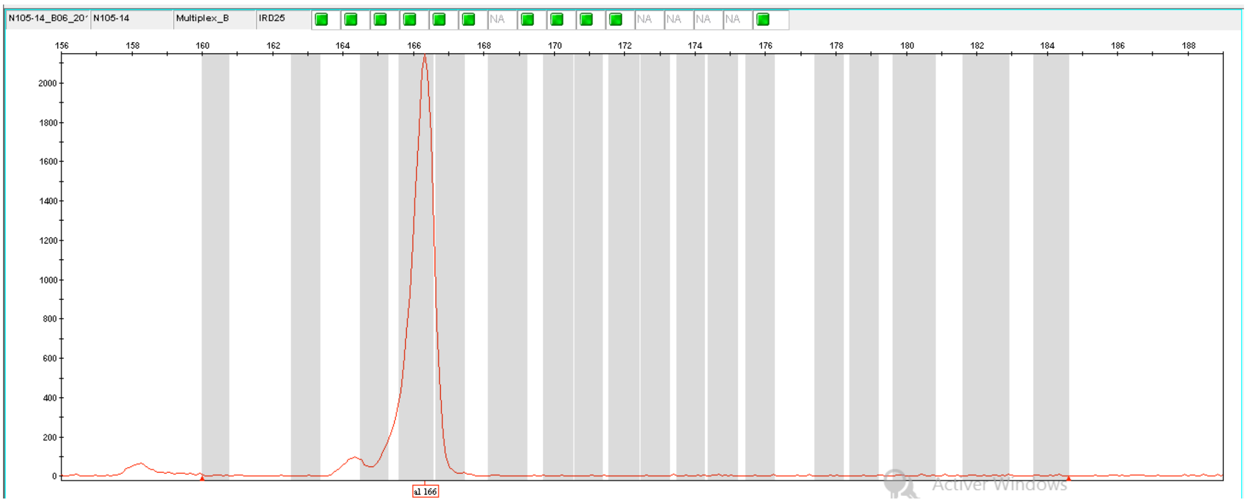

10: Microsatellite marker *PGIRD25* profile on sample N_105-14

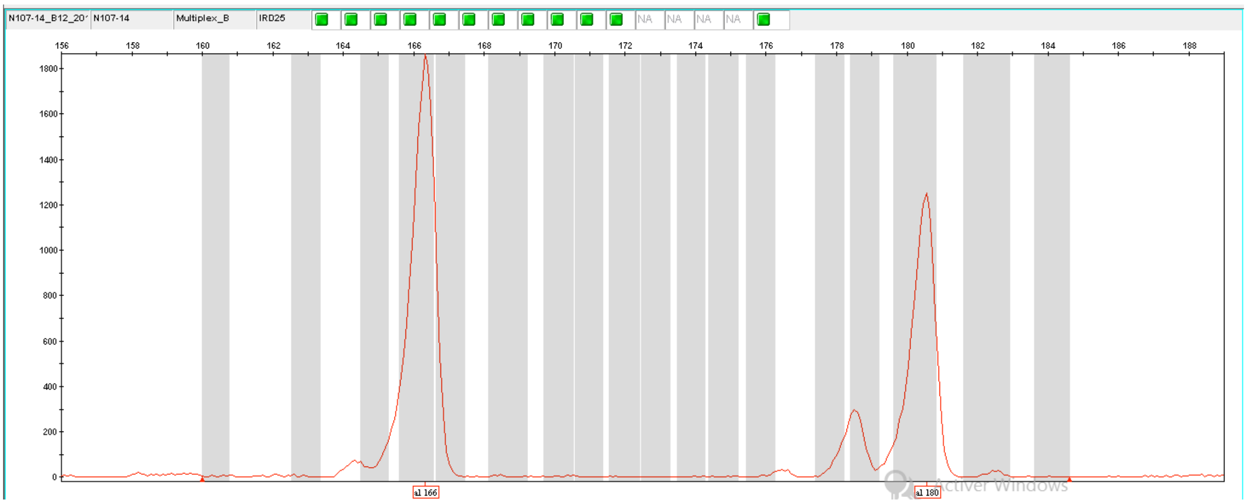

11: Microsatellite marker *PGIRD25* profile on sample N_107-14

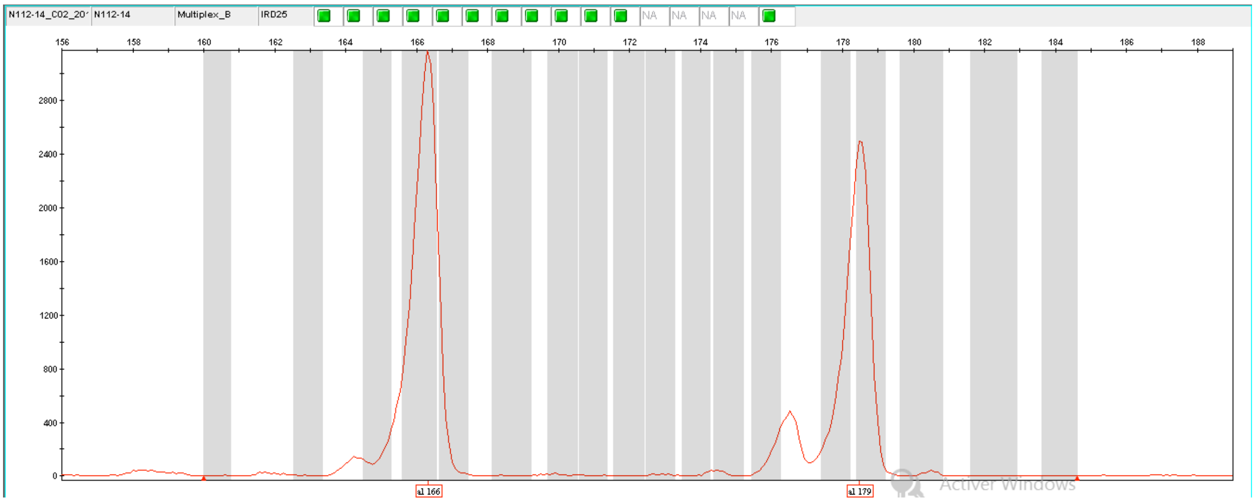

12 : Microsatellite marker *PGIRD25* profile on sample N_112-14

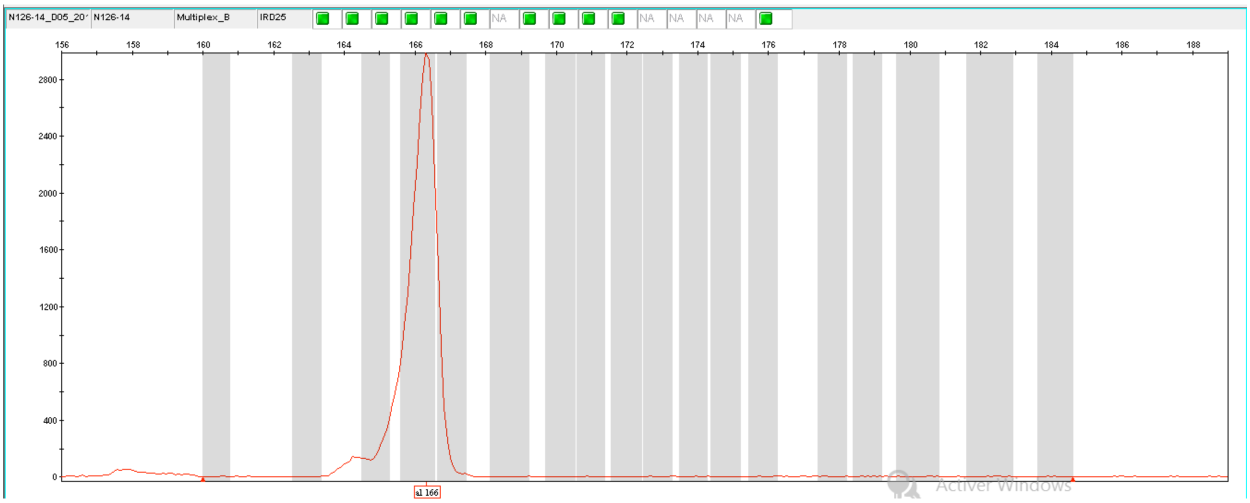

13: Microsatellite marker *PGIRD25* profile on sample N_126-14

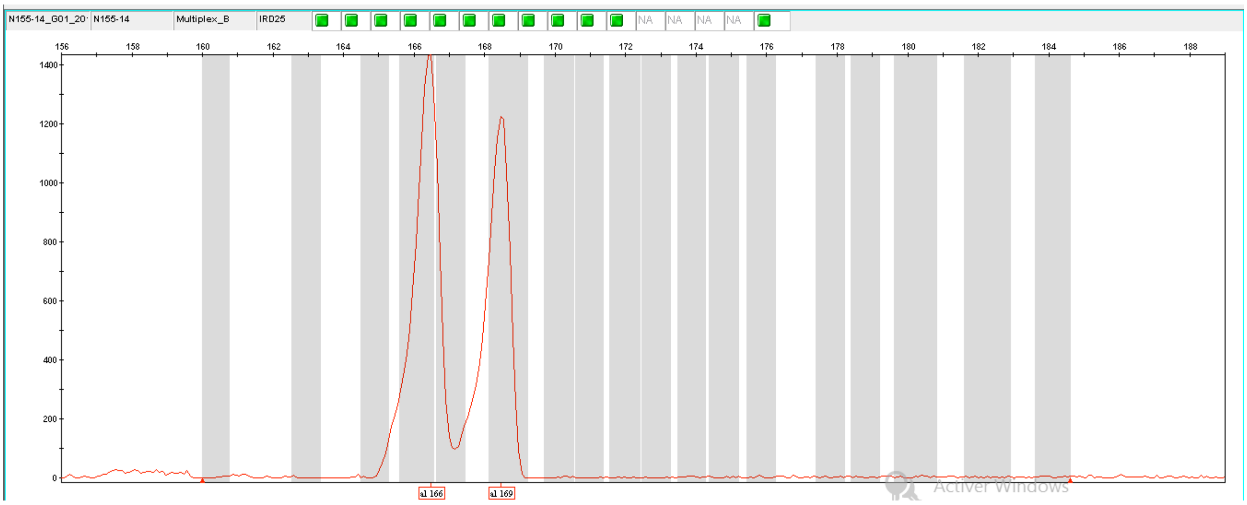

14: Microsatellite marker *PGIRD25* profile on sample N_155-14

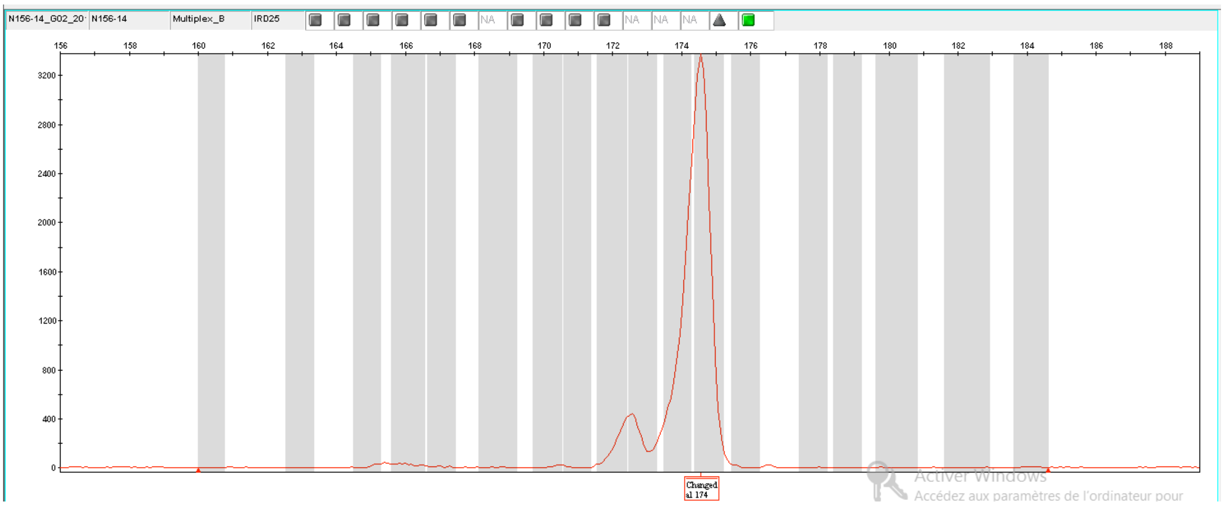

15: Microsatellite marker *PGIRD25* profile on sample N_156-14

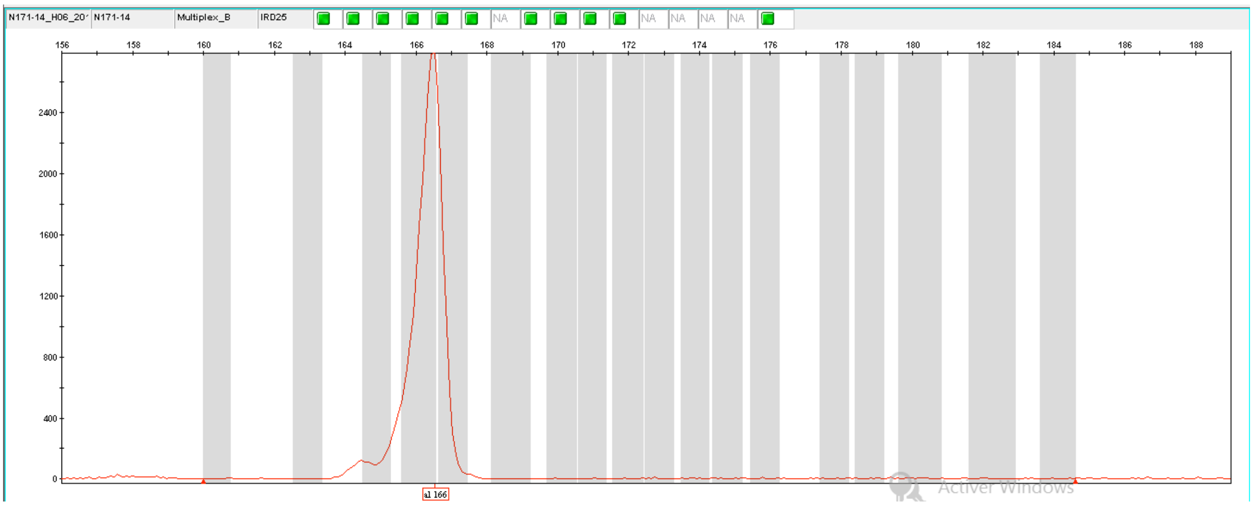

16: Microsatellite marker *PGIRD25* profile on sample N_171-14

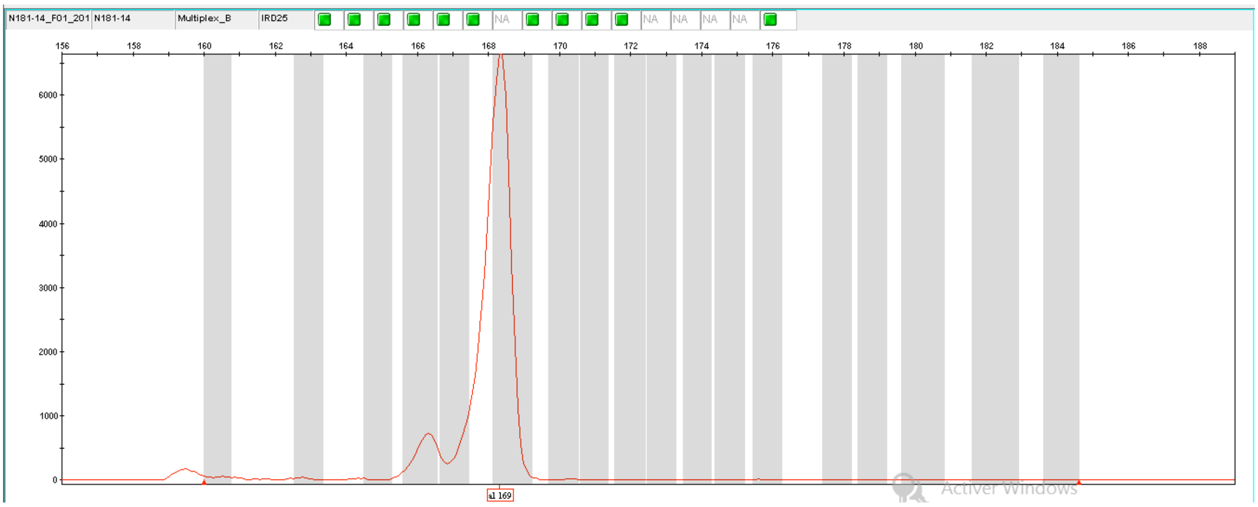

17: Microsatellite marker *PGIRD25* profile on sample N_181-14

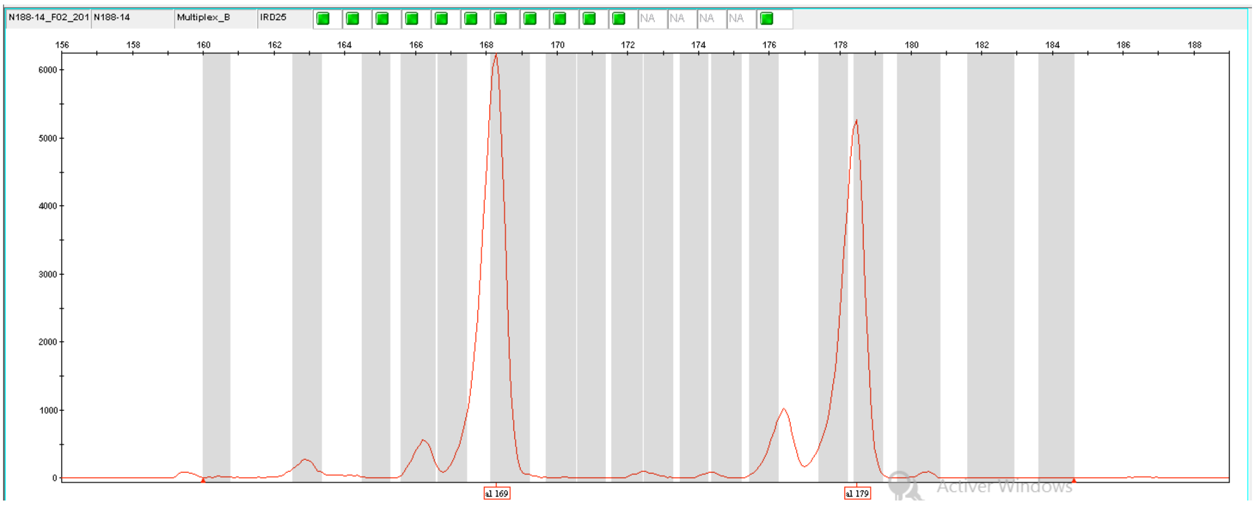

18: Microsatellite marker *PGIRD25* profile on sample N_188-14

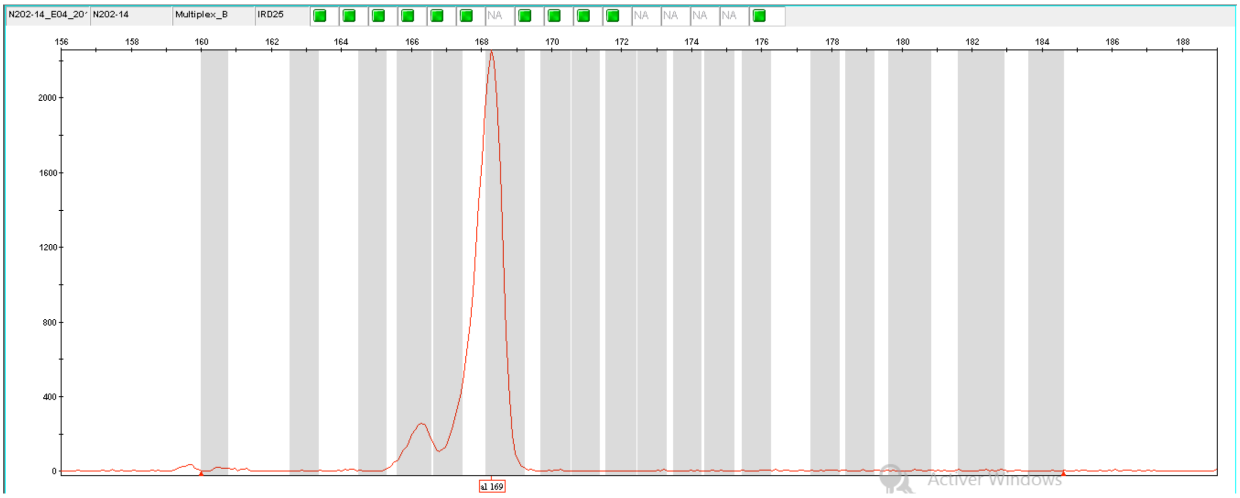

19: Microsatellite marker *PGIRD25* profile on sample N_202-14

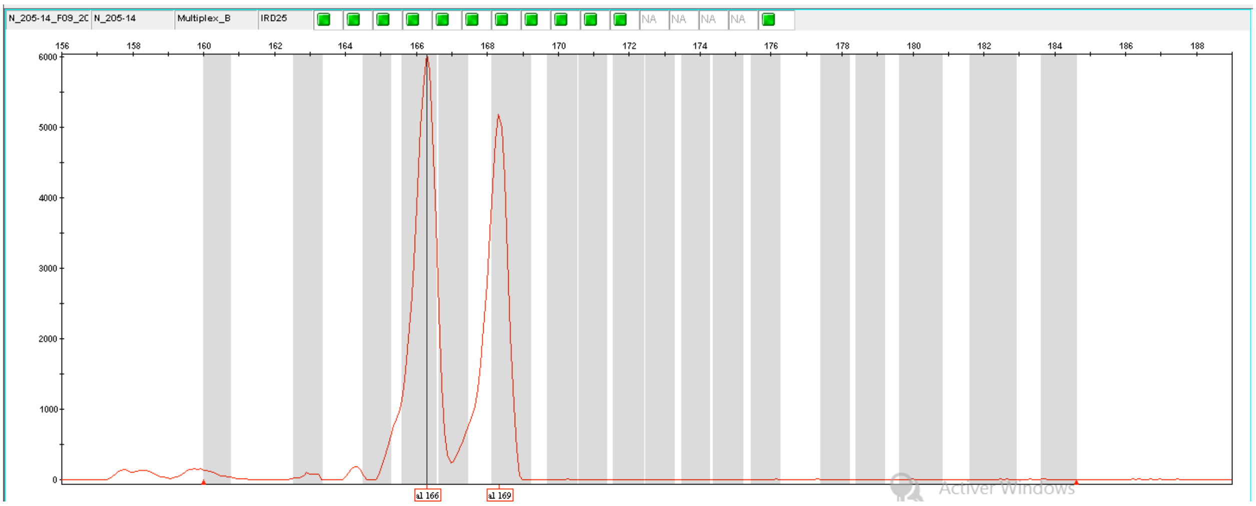

20: Microsatellite marker *PGIRD25* profile on sample N_205-14

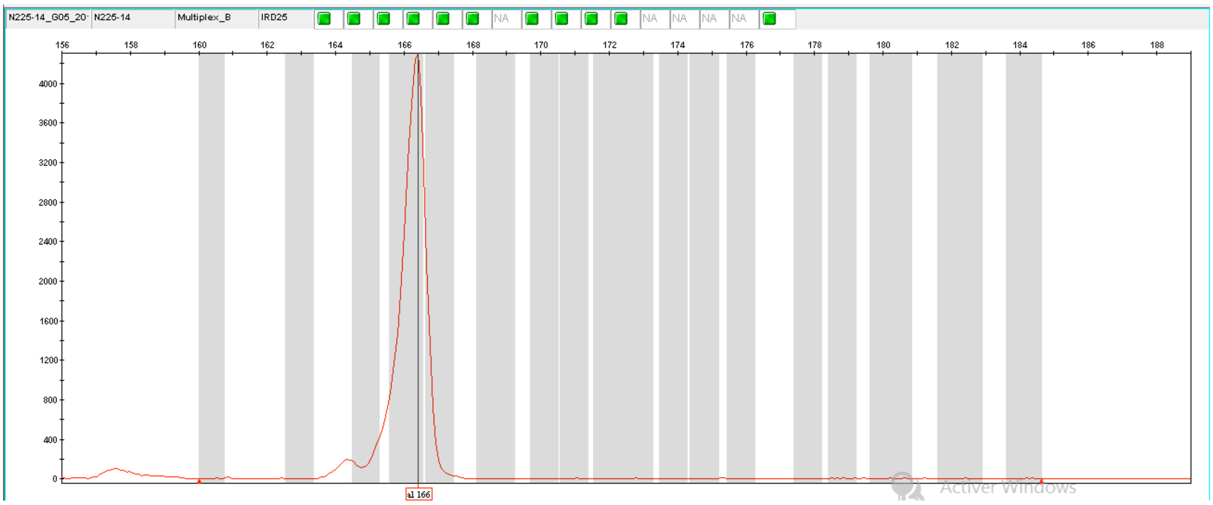

21: Microsatellite marker *PGIRD25* profile on sample N_225-14

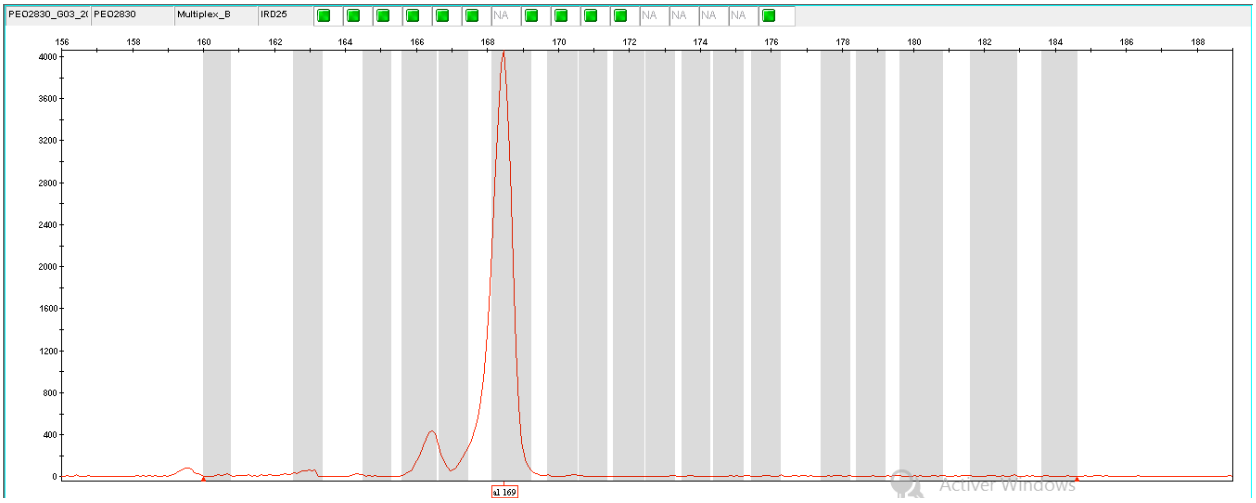

22: Microsatellite marker *PGIRD25* profile on sample PE0_2830

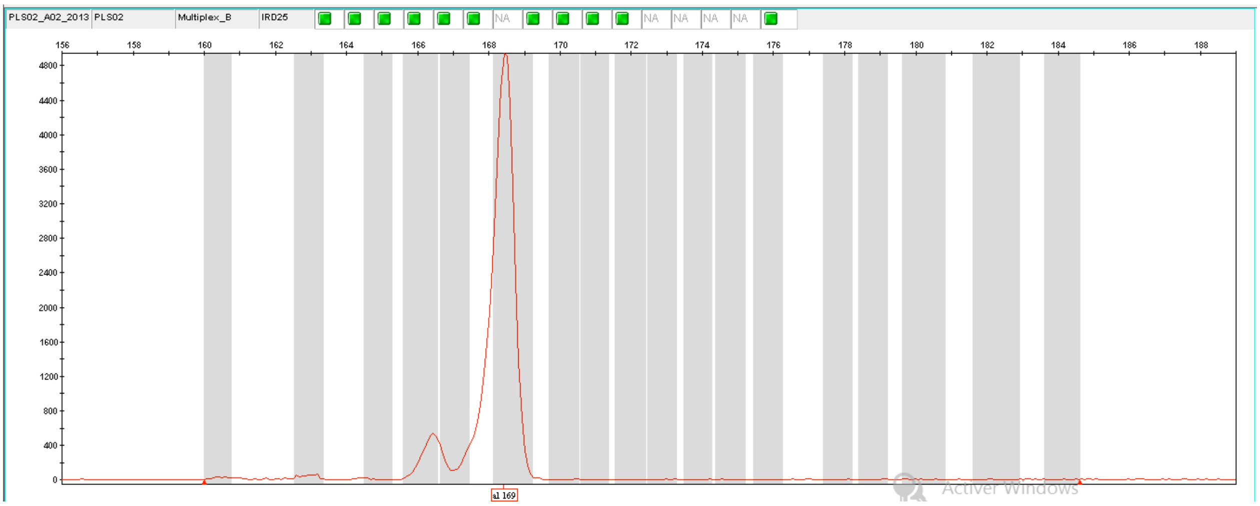

23: Microsatellite marker *PGIRD25* profile on sample PLS_02

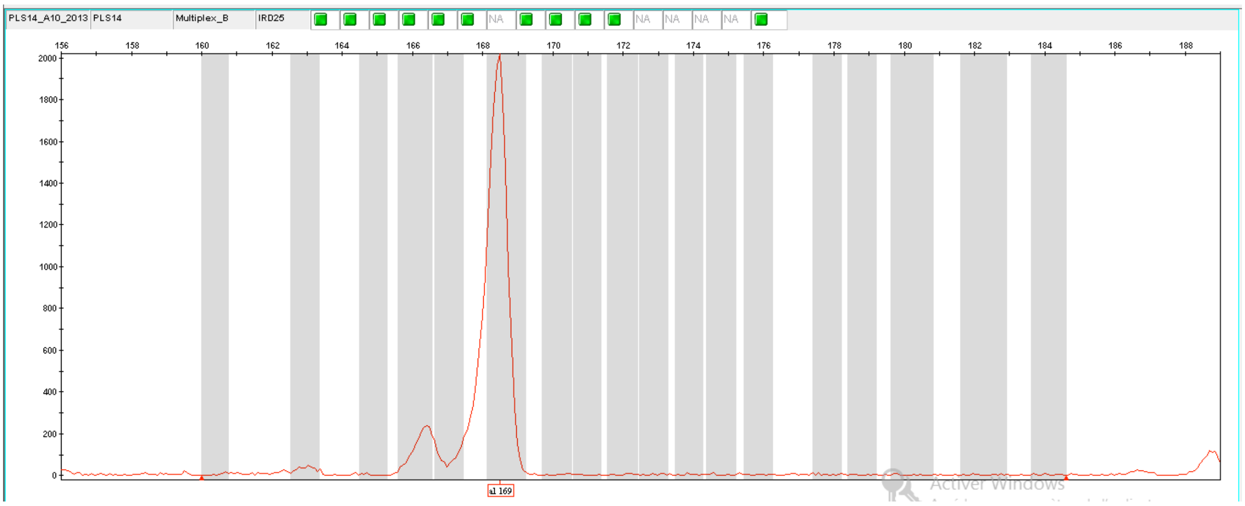

24: Microsatellite marker *PGIRD25* profile on sample PLS_14

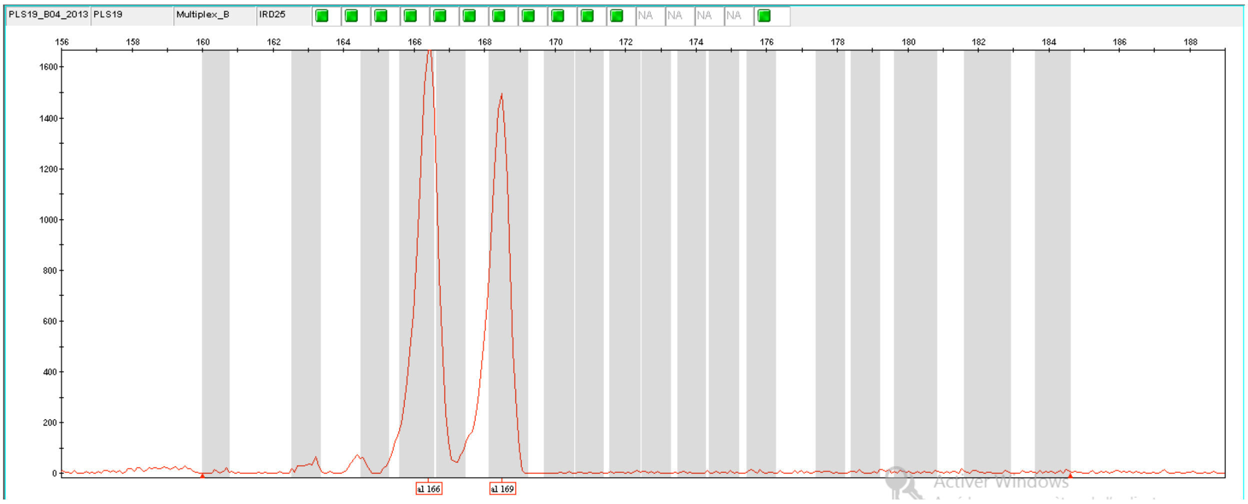

25: Microsatellite marker *PGIRD25* profile on sample PLS_19

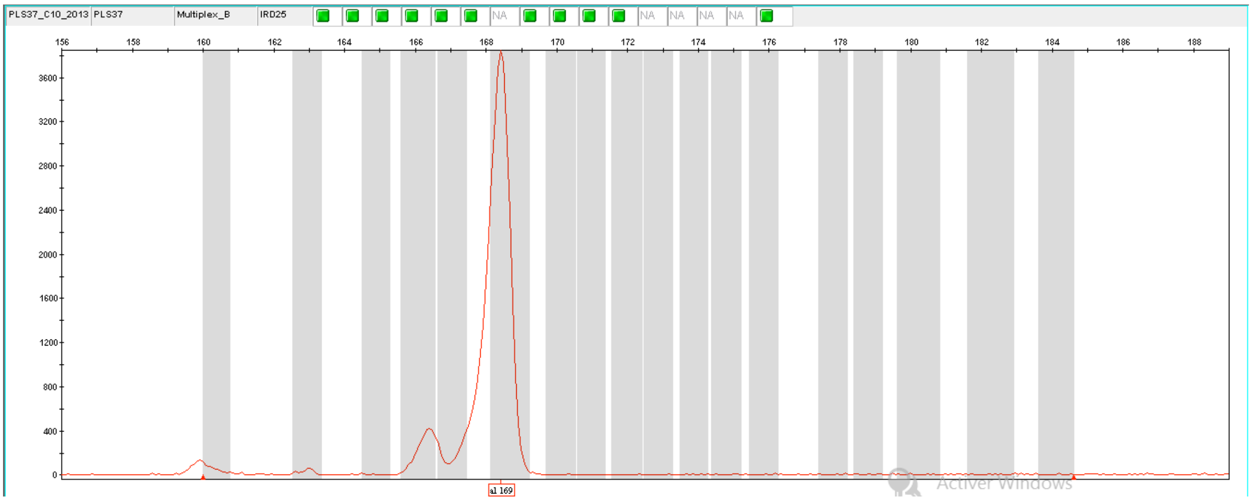

26: Microsatellite marker *PGIRD25* profile on sample PLS_37

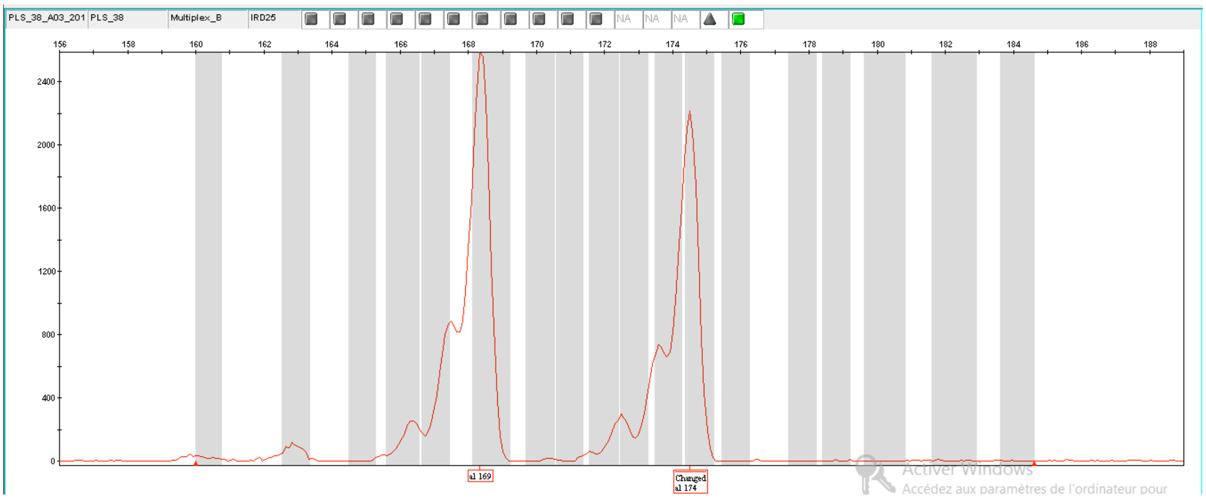

27: Microsatellite marker *PGIRD25* profile on sample PLS_38

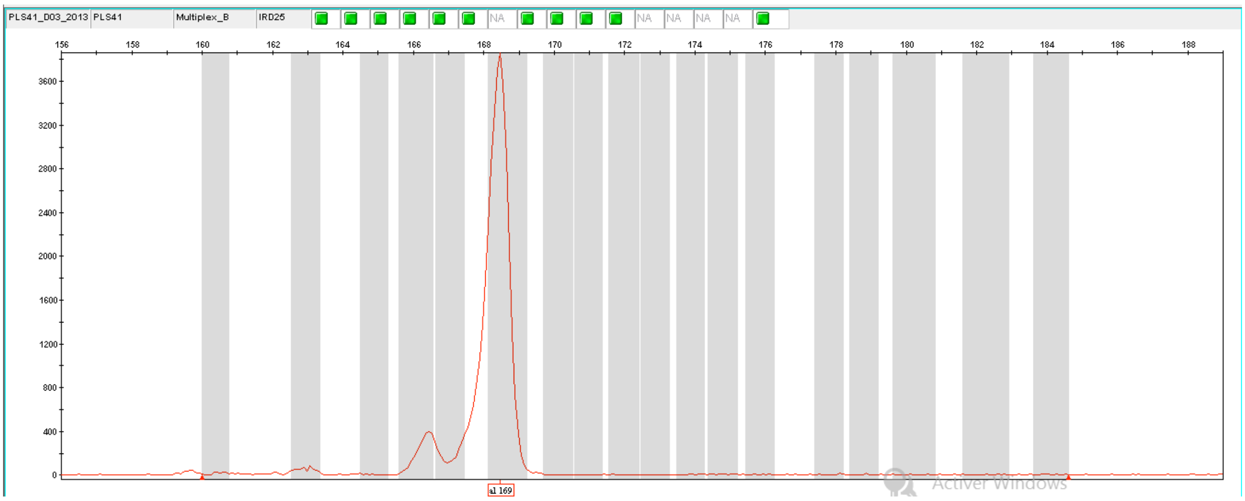

28: Microsatellite marker *PGIRD25* profile on sample PLS_41

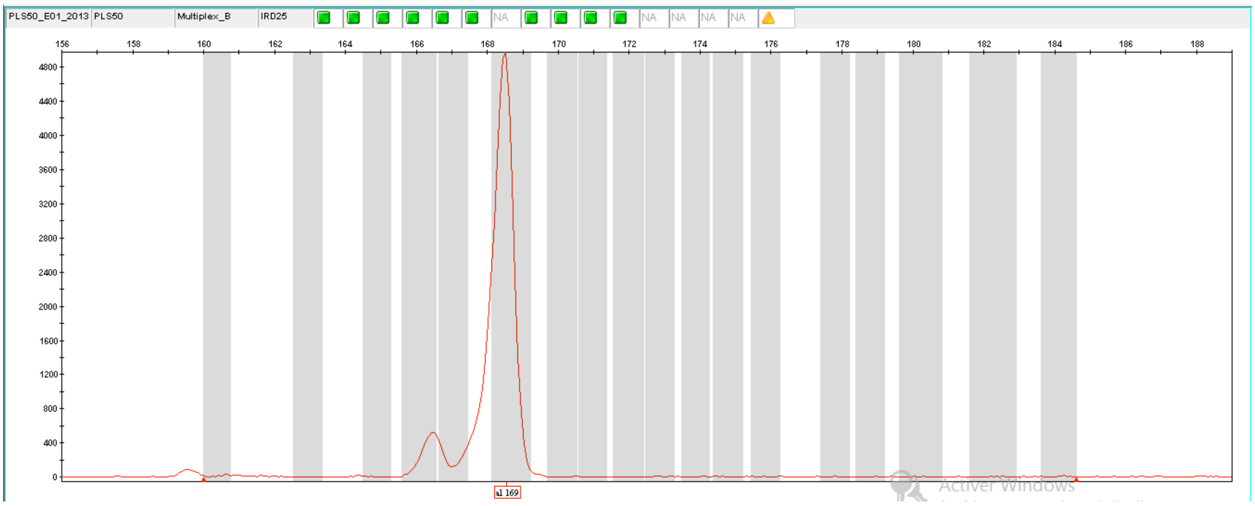

29: Microsatellite marker *PGIRD25* profile on sample PLS_50

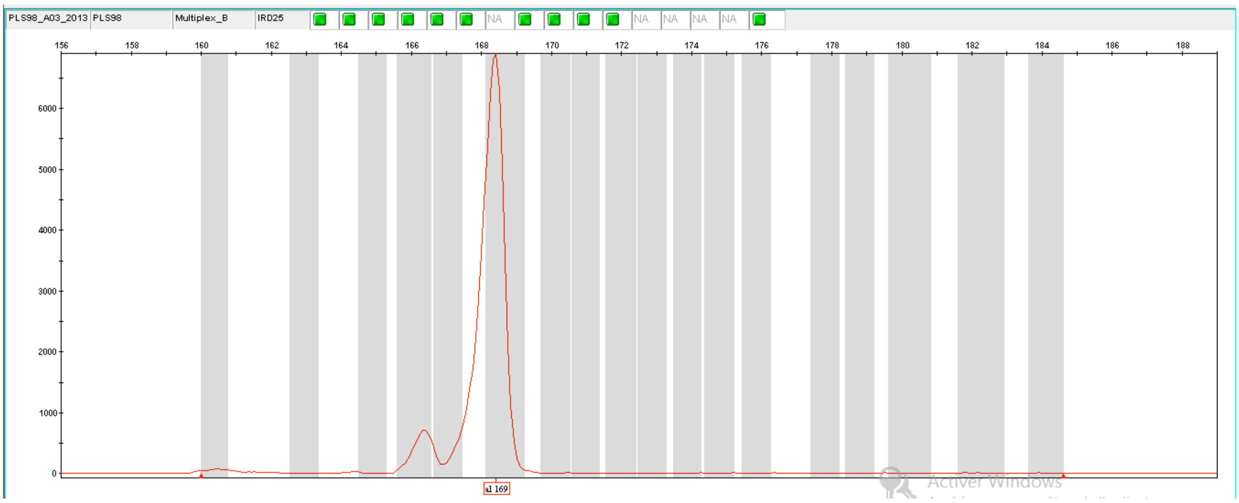

30: Microsatellite marker *PGIRD25* profile on sample PLS_98

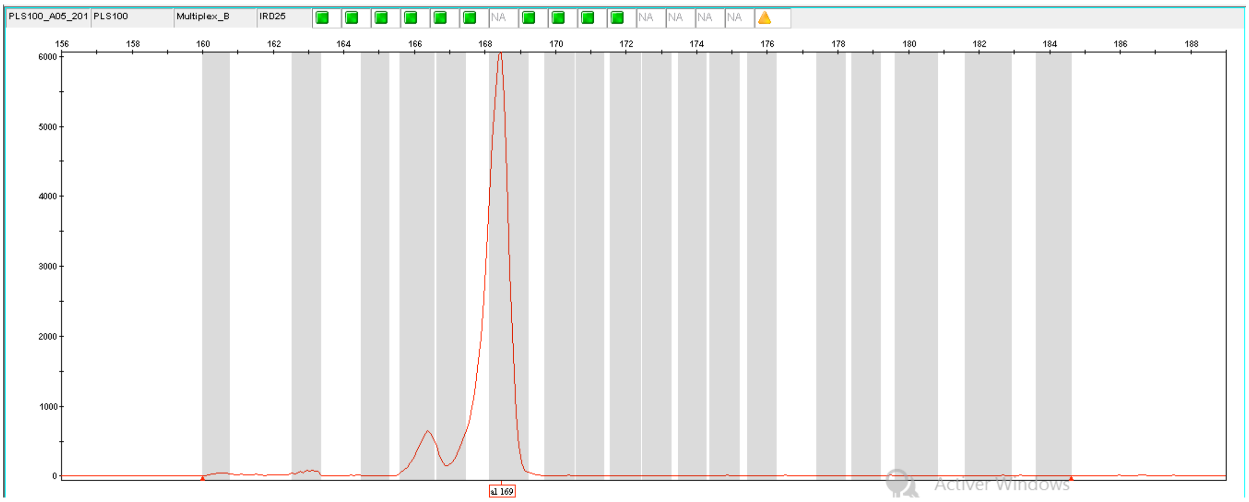

31: Microsatellite marker *PGIRD25* profile on sample PLS_100

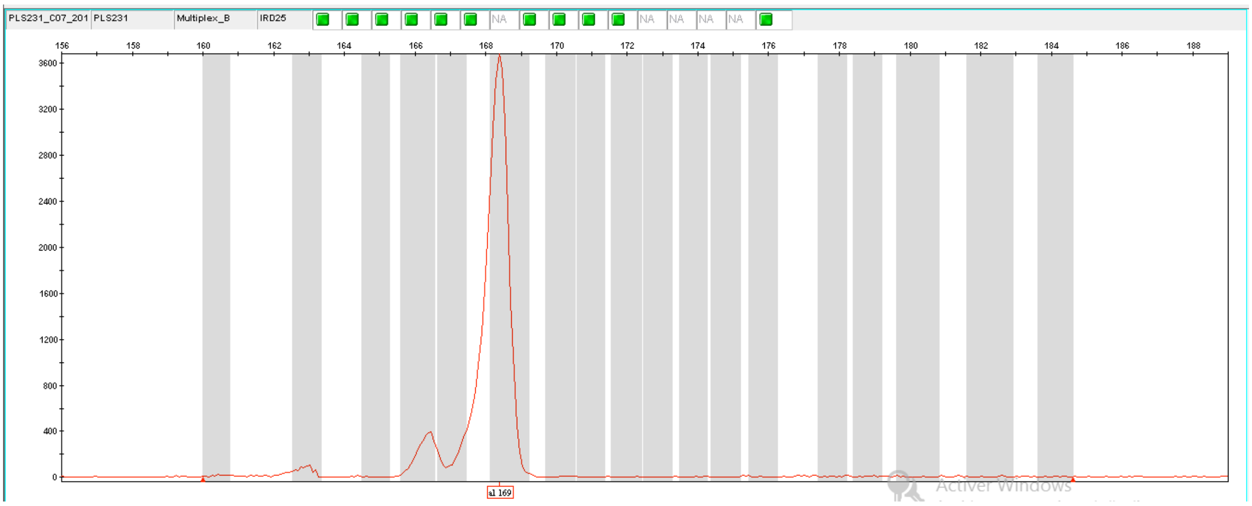

32: Microsatellite marker *PGIRD25* profile on sample PLS_231

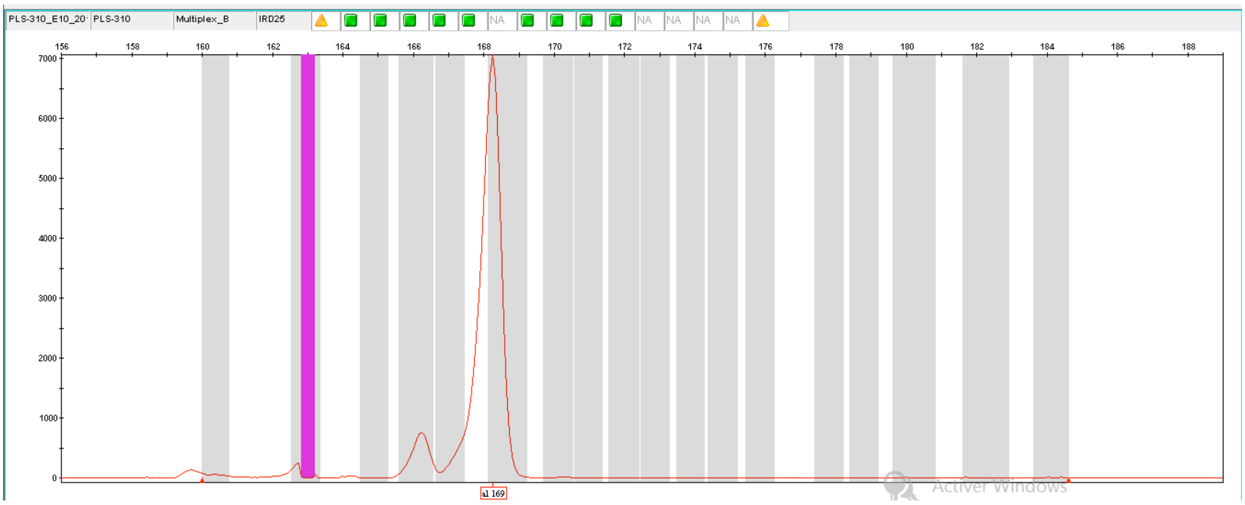

33: Microsatellite marker *PGIRD25* profile on sample PLS_310

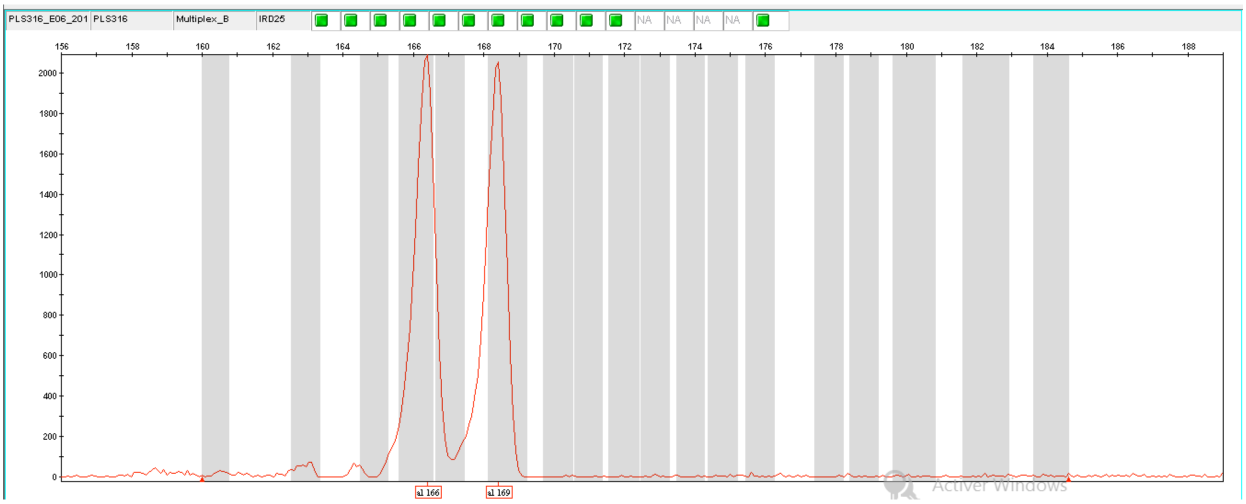

<u>34:</u> Microsatellite marker *PGIRD25* profile on sample PLS_316

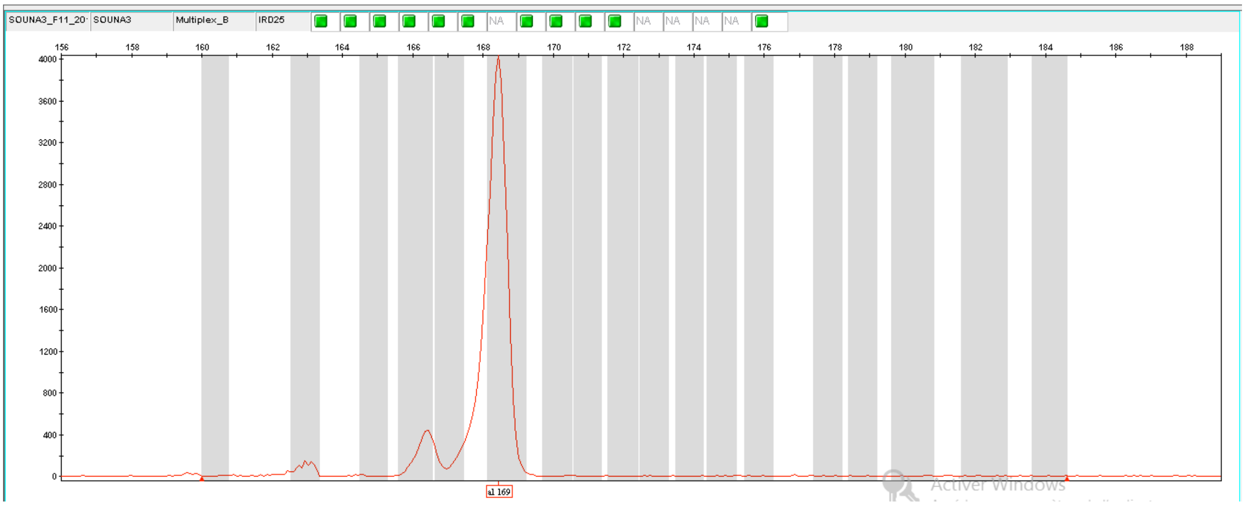

<u>35:</u> Microsatellite marker *PGIRD25* profile on sample SOUNA_3

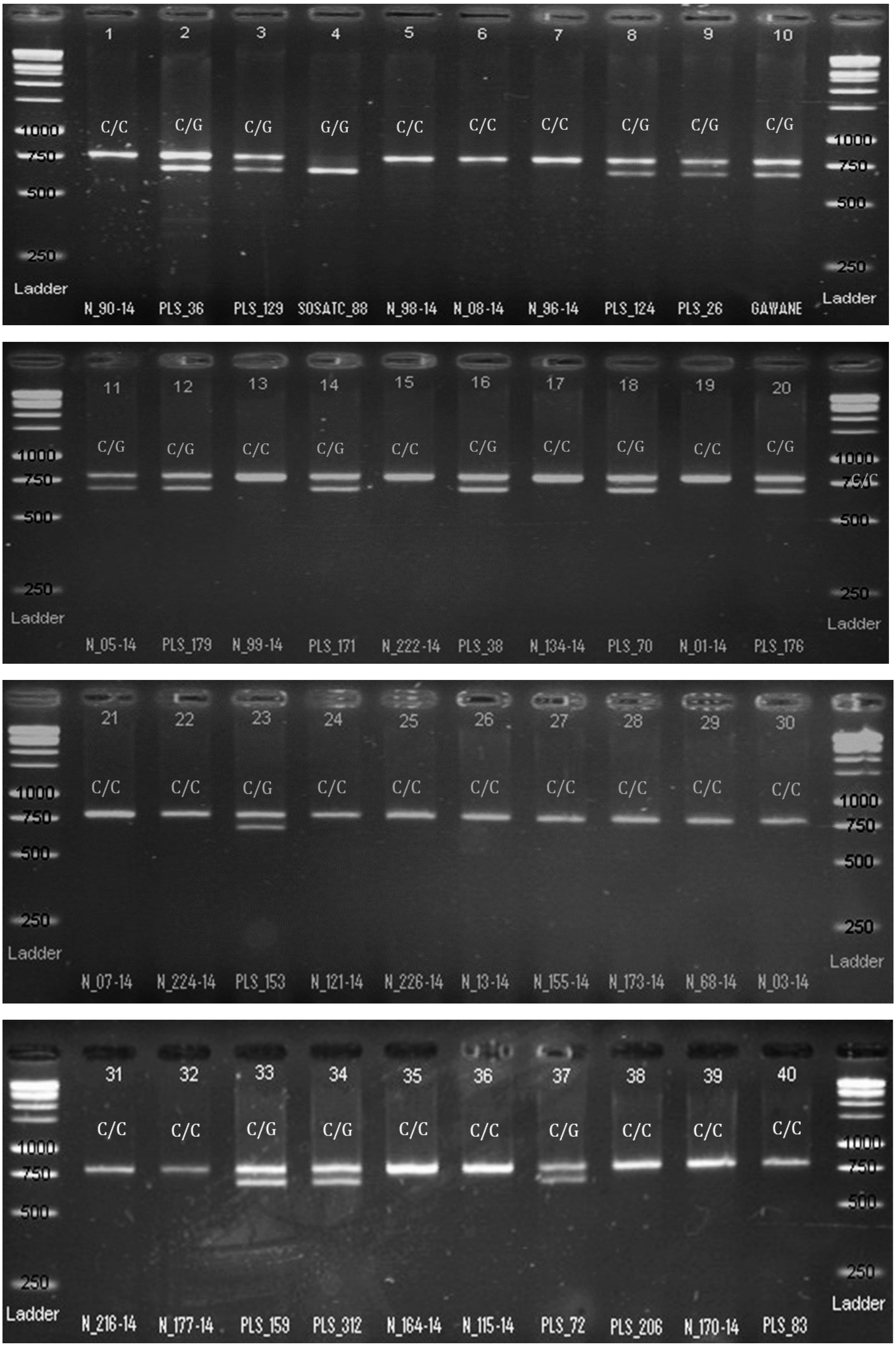

Figure S4. Genotype profiles for *PgPHYC* among accessions. A C/G SNP at 697bp on the 799pb PCR fragment is cleaved by PvuII restriction enzyme and therefore accessions scored as C/C, G/G and C/G. Only 40 accessions are scored here. The other scores are given in Table S1.

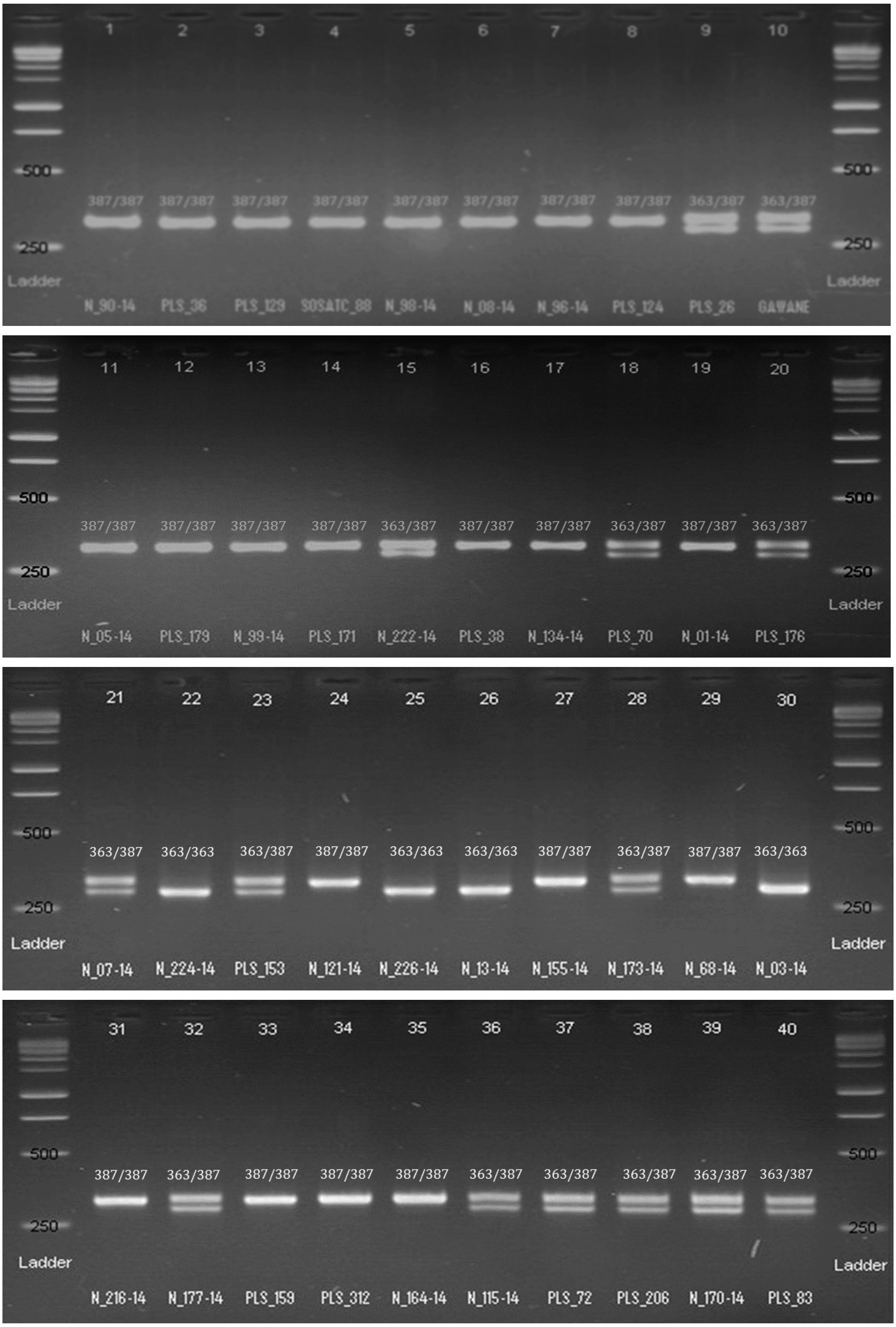

Figure S5. Genotype profiles for *PgMADS11* among accessions. An indel polymorphism of 24 bp distinguishes both alleles among individuals. Only 40 accessions are scored here. The other scores are given in Table S1.
